# Author Correction: Commensal yeast promotes *Salmonella* Typhimurium virulence

**DOI:** 10.1038/s41586-026-10518-3

**Published:** 2026-04-24

**Authors:** Kanchan Jaswal, Olivia A. Todd, Roberto C. Flores Audelo, William Santus, Saikat Paul, Ciera M. Duffy, Edward T. Eshoo, Manmeet Singh, Jian Miao, David M. Underhill, Brian M. Peters, Judith Behnsen

**Affiliations:** 1 Department of Microbiology and Immunology, University of Illinois Chicago, Chicago, IL USA; 2 Department of Biological Sciences, University of Illinois Chicago, Chicago, IL USA; 3 Department of Clinical Pharmacy and Translational Science, College of Pharmacy, University of Tennessee Health Science Center, Memphis, TN USA; 4 Department of Pathology, University of Illinois Chicago, Chicago, IL USA; 5 Pharmaceutical Sciences Program, College of Graduate Health Sciences, University of Tennessee Health Science Center, Memphis, TN USA; 6 Department of Biomedical Sciences, Cedars-Sinai Medical Center, Los Angeles, CA USA; 7 F. Widjaja Inflammatory Bowel and Immunobiology Research Institute, Cedars-Sinai Medical Center, Los Angeles, CA USA; 8 Department of Microbiology, Immunology, and Biochemistry, College of Medicine, University of Tennessee Health Science Center, Memphis, TN USA

**Keywords:** Fungi, Bacterial pathogenesis, Microbiome, Bacterial host response

Correction to: *Nature* 10.1038/s41586-025-09415-y Published online 3 September 2025

In the version of this article initially published, there was an error in the first paragraph of the “*C. albicans* enhances *Salmonella* virulence” section, where in the text now reading “Faeces of mice from cage 1,” “cage 1” appeared as “cage 3.” The legends to Fig. 5b,d, originally reporting significance derived from two-tailed unpaired Student’s *t*-tests, have been amended to note that Mann Whitney *U* tests were used. In the significance bars in Fig. 5b, “*P* = 0.0107” and “*P* = 0.0274” now replace the original “*P* = 0.0202” and “*P* = 0.0343.” In Extended Data Fig. 1a, dots depicting mice in cage 1 before infection were missing, and the legend has been amended to read “Data are median with range” from the original “Data are geometric mean ± s.d.” The text and figures have been amended in the HTML and PDF versions of the article.

