# Supplementary material for: Commensal yeast promotes Salmonella Typhimurium virulence
Source: Nature. 2025 Sep 3;645(8082):1002–10. doi: 10.1038/s41586-025-09415-y (PMC12460169; doi:10.1038/s41586-025-09415-y)
Supplement: Supplementary file 1 — This file contains Supplementary Methods, Supplementary Tables 14–16, Supplementary Figures and Supplementary References. [file 41586_2025_9415_MOESM1_ESM.pdf]

nature portfolio

https://doi.org/10.1038/s41586-025-09415-y

Supplementary information

Commensal yeast promotes *Salmonella* Typhimurium virulence

In the format provided by the authors and unedited

Supplementary Materials and Methods

C. albicans strain construction.

Construction of a C. albicans constitutive GFP reporter: Strain SC5314 was transformed with SfiI-linearized vector pDUP3-GFPy via the lithium acetate method as described [1]. Transformants were selected on YPD plates containing NAT (200 mg/l). Integration of the cassette was confirmed with primer pairs NEUT5LAMPF + NAT1INTF and NEUT5LAMPR + tADH1AMPR-SpeI (Table S16). Positive fluorescence signal was confirmed by epifluorescence microscopy using a 488 nm laser and corresponding filters.

Construction of arg4Δ/Δ strain using CRISPR-Cas9 mediated gene editing system: The *arg4*Δ/Δ strain was constructed by amplifying linearized *SAT1*-flipper and *CaHygB*-flipper plasmids with ARG4CC9KO-F + ARG4CC9KO-R primers to make disruption repair templates bearing nourseothricin (NAT) and hygromycin B (HygB) resistance cassettes, respectively (Table S15, S16). Before the transformation, *C. albicans* competent cells were prepared by treating freshly grown cells with 1X Tris-EDTA and 0.1 M lithium acetate (pH 7.5) for 1 h at 37°C followed by 25 mM dithiothreitol for an additional 30 min. Cells were washed with sterile water, followed by washing with 1 M sorbitol and resuspended in residual sorbitol. Cells were mixed with *in vitro* assembled ribonucleoprotein (RNP) complexes containing universal tracrRNA, Cas9 protein (2 mg), *ARG4* gene-specific guide RNAs [crARG4up (5`-GATTTATAGGAATCACTTTT), and crARG4down (5`-AACAGCAGCTTTCAAGAATA) (Table S16)], and 1 µg of each PCR-generated repair template. The transformation was performed with a single pulse at 1.8 kV using the Gene Pulser Xcell Electroporation System (Bio-Rad). Cells were immediately incubated at 30°C for 4-6 hours in YPD medium and plated on YPD agar containing NAT (200 mg/l) and HygB (600mg L^{-1}) (GoldBio). Selected colonies were transferred to yeast-peptone medium with 2% maltose (YPM) for overnight culture to induce cassette excision. Cells were then selected on YPD agar containing NAT (25 mg/l) and HygB (75 mg L^{-1}). Cassette excision and expected auxotrophy was confirmed by growing transformants on YPD, YPD containing NAT (200 mg L^{-1}), and YPD containing HygB (600 mg L^{-1}) or yeast nitrogen base (YNB) without amino acids and YNB without amino acids containing 100 mg ml^{-1} arginine, respectively. Targeted integration and *ARG4* deletion was confirmed by PCR via amplification of genomic DNA using the primers ARG4DETF + ARG4DETR, ARG4INTF + FLPINTR, and ARG4INTR + FLPINTF as described (Table S16) [1,2].

Construction of arg4Δ/Δ+ARG4 (ARG4 revertant) strain: The *arg4*Δ/Δ+*ARG4* strain was constructed using overlap extension PCR as described [2]. The integration cassette consisted of PCR-amplified *5'* (Neut5homology-pDIS3F + Nt5ADH1upUNIVOL-R) and 3' (ADH1t-UNIVOL-F + NEUT5homology-pDIS3R) flanking *NEUT5* homology arms using linearized plasmids pDIS3-tADH1. The 3' fragment also contained the Ca*ADH1* terminator and NAT1 encoding for NAT resistance. The *ARG4* promoter and ORF were amplified from *C. albicans* gDNA using PrARG4OL-F + tARG4OL-R (Table S16). Overlap extension of the amplified products was performed as described [2]. The overlap product was used as the template in a subsequent PCR reaction using NEUT5homology-pDIS3F + NEUT5homology-pDIS3R primers to make the repair template. The transformation was performed using the CRISPR-Cas9 protocol above, except that crNEUT5pDISup (5`-GCTCGGAGGAGGCTCCCCAA) crRNA was used during RNP assembly and introduced into *arg4*Δ/Δ. Phenotypic confirmation of the transformed cells was confirmed by restoration of growth on YNB without amino acids. The genotype was confirmed by using primers NAT1INTF + NEUT5LAMPR and ARG4DETF + ARG4DETR (Table S16) [1,2].

Construction of SopB expressing C. albicans: Vector CIpSATtetTrans contains the tetracycline-regulatable (TR) promoter *tetO* and TR transactivator [3]. To construct a doxycycline-repressible SopB strain, the CIpSATtetTrans vector and the ADH1 terminator synthetic DNA preceded by a new multiple cloning site (MCS-tADH1) were restriction digested with HindIII and MluI, followed by ligation and transformation into *E. coli* DH5α. Transformants were screened on LB agar plates containing ampicillin. Furthermore, the transformed plasmid named CIpSATtetTransMCStADH1 was verified for integrity using restriction digestion and sequencing (Plasmidsaurus). *C. albicans*-optimized STm *sopB* synthetic DNA (CaOptSTSopB) was PCR-amplified using SynthGene-F + SynthGene-Rv2 primers. This PCR product and plasmid CIpSATtetTransMCStADH1 were digested with HindIII and EagI, subsequently ligated, and transformed into *E. coli* DH5α to create plasmid CIpSATtetTransMCStADH1-SopB. Plasmid sequence was verified as described above. The lithium acetate protocol was used as described previously with some modifications to create *C. albicans* strains containing doxycycline-repressible *sopB* or empty vector at the *RPS1* (*i.e., RP10*) locus[4,5]. In brief, 25 µl of overnight culture was mixed with 5 ml YPD and incubated at 30°C with continuous shaking for 6 hours. After washing with sterile water, cells were resuspended in 1 ml 1X TELiAc buffer (100 mM LiAc, 1X TE). After centrifugation, the cells were resuspended in 50 µl 1X TELiAc, and 1 µg of StuI-linearized CIpSATtetTransMCStADH1-SopB or CIpSATtetTransMCStADH1 was added. Additionally, 5 µl of single-stranded salmon sperm carrier DNA and 300 µl of 40% PEG were

added to the suspension. The transformation mix was incubated for 30 minutes at 30°C with gentle agitation every 10 minutes. Cells were then heat-shocked at 42°C for 15 minutes and allowed to recover in YPD at 30°C for 4 h with continuous shaking. Cells were transferred to YPD agar containing 200 mg/l NAT and 10 mg/l doxycycline for selection and transgene repression, respectively. Resulting colonies were further confirmed genotypically by PCR using primers CaSopBDETF + CaSopBDETR and ADH1term-F-ClaI + RP10AMPR (Table S16).

ELISA for cytokine quantification. Blood was collected from mice via cardiac puncture in serum collection tubes from BD Microtainer® and centrifuged at 2000g for 10minutes to separate serum. Cytokine quantification was performed using the uncoated ELISA kit (Invitrogen) for IL-1β, TNFα, and IFNγ. Depending on serum availability, samples were diluted in the range of 1:2 to 1:5 in 1X ELISA Diluent (prepared per kit instructions). Dilutions were accounted for in data analysis. Absorbance at 450nm was measured in the BioTek Synergy HTX multimode microplate reader.

Histopathology. Tissue sections were fixed with formalin and embedded in paraffin wax. Embedding was performed by the Research Histology core at the University of Illinois Chicago. The tissue was then sectioned via microtome and transferred to slides. Before staining, deparaffinization was performed. The tissue slides were immersed in xylene for 10 min, 100% ethanol for 10 min, 90% ethanol for 2 min, 70% ethanol for 2 min, and PBS for 5 min. The tissue slides were then stained with hematoxylin for 30 seconds, washed with tap water, then stained with eosin for 10 min. Slides were dehydrated by immersion in serial increases of ethanol concentrations (50%-100%), then immersed in xylene. Coverslips were then mounted to the tissue slides and allowed to dry.

Tissue sections were scored for pathology by a board-certified pathologist in a blinded fashion following an approach established by Barthel and colleagues[6] as summarized below. Submucosal edema was scored as follows: 0 = no pathological changes; 1 = mild edema (submucosa accounts for <50% of the diameter of the entire intestinal wall [tunica muscularis to epithelium]); 2 = moderate edema; the submucosa accounts for 50 to 80% of the diameter of the entire intestinal wall; and 3 = profound edema (the submucosa accounts for >80% of the diameter of the entire intestinal wall).

Polymorphonuclear granulocytes (PMN) in the lamina propria were enumerated in 10 high-power fields (x400 magnification), and the average number of PMN/high-power fields was

calculated. The scores were defined as follows: 0 = <5 PMN/high-power field; 1 = 5 to 20 PMN/high-power field; 2 = 21 to 60/high-power field; 3 = 61 to 100/high-power field; and 4 = >100/high-power field. Transmigration of PMN into the intestinal lumen was consistently observed when the number of PMN was >60 PMN/high-power field.

The average number of goblet cells per high-power field (magnification, x400) was determined from 10 different regions of the cecal epithelium. Scoring was as follows: 0 = >28 goblet cells/high-power field (magnification, x400); 1 = 11 to 28 goblet cells/high-power field; 2 = 1 to 10 goblet cells/high-power field; and 3 = <1 goblet cell/high-power field.

Epithelial integrity was scored as follows: 0 = no pathological changes detectable in 10 high power fields (x400 magnification); 1 = epithelial desquamation; 2 = erosion of the epithelial surface (gaps of 1 to 10 epithelial cells/lesion); and 3 = epithelial ulceration (gaps of >10 epithelial cells/lesion).

Two independent scores for submucosal edema, PMN infiltration, goblet cells, and epithelial integrity were averaged for each tissue sample. The combined pathological score for each tissue sample was determined as the sum of these averaged scores. It ranges between 0 and 13 arbitrary units and covers the following levels of inflammation: 0 intestine intact without any signs of inflammation; 1 to 2 minimal signs of inflammation; 3 to 4 slight inflammation; 5 to moderate inflammation; and 9 to 13 profound inflammation.

P22 transduction to generate IR715 Δ*STM4351*. Δ*STM4351* strain was constructed by P22 transduction using the P22 *HT105/1 int-201* bacteriophage. A culture of the STm 14028 Δ*STM4351* strain was infected with P22 HT105/1 int-201 for 8 h at 37˚C. The phage was isolated by the centrifugation of the infected culture, and chloroform was added to the collected supernatant. Bacteriophage transduction was performed by plating 200 μL of a 16 h STm IR715 WT culture and 1–20 μL of the isolated P22 phage onto LB agar with kanamycin (0.1 mg/mL). Plates were incubated at 37˚C overnight, and the resulting colonies of transductants were cross-streaked against the P22 H5 phage on Evans blue-Uranine (EBU) agar to isolate phage-free true lysogens. Transductants were confirmed using PCR primer pairs: STM4351F + STM4351R (Table S16).

Growth curve of Δ*STM4351*. 200μL of $2x10^6$ cell/ml of culture was added in triplicate to a flat-bottom, clear, microwell plate. The growth experiment was performed at 37°C with slow orbital

shaking for 30 seconds every 15 minutes in a BioTek Synergy HTX multimode microplate reader. OD_{600} was measured hourly.

Supplementary Tables

Table S14. Strains used in this study.

**Strain name** (as used in this study)	**Relevant genotype/ feature**	**Source**
Salmonella enterica serovar Typhimurium strains		
STm	IR715; ATCC 14028 with spontaneous Nal^R derivative	ATCC
STm (Mouse experiments)	IR715 + pHP45Ω	Vladimir E. Diaz-Ochoa
Δ*fim*	EHW2; IR715 Δ*fimAICDHF*	Weening et al 2005[7]
Δ*bcf*	EHW1; IR715 *bcfABCDEFG::kan*	Weening et al 2005[7]
Δ*invA*	BL212; IR715 Δ*invA*	Devlin et al 2022[8]
Δ*spiB*	SPN450; IR715 *spiB::*KSAC	Manuela Raffatellu et al 2009[9]
Δ*invA*/Δ*spiB*	SPN452; IR715 *invA::tetRA spiB*::KSAC	Manuela Raffatellu et al 2009[9]
STm (fluorescence microscopy)	IR715 + pFPV-*mCherry*	This study
hilA-*lacZ*	JS749; attλ::pDX1::*hilA'*-*lacZ*	Lin et al 2008[10]
Δ*sipA* Δ*sopABDE2*	ZA21; IR715 Δ*sipA* Δ*sopABDE2*	Zhang et al 2002[11]
Δ*sipA*	ZA10; IR715 Δ*sipA*	Zhang et al 2002[11]
Δ*sopABDE2*	ZA20; IR715 Δ*sopABDE2*	Zhang et al 2002[11]
Δ*sopBE2*	ZA16; IR715 Δ*sopBE2*	Zhang et al 2002[11]
Δ*sopB*	ZA15; IR715 Δ*sopB*	Zhang et al 2002[11]
SL1344		Hoiseth S.K et al 1981[12]
SL1344 Δ*sopB*	SL1344 Δ*sigDE*	Knodler et al 2006[13]
SL1344 Δ*sopB* + p*sopB*	SL1344 Δ*sigDE* pWSKDE (pWSK29+*sigDE*)	Knodler et al 2009[14]
SL1344 Δ*sopBE*	SL1344 Δ*sopB*Δ*sopE*::*aphT*	Cooper et al 2011[15]

**Strain name** (as used in this study)	**Relevant genotype/ feature**	**Source**
SL1344 Δ*sopBE2*	SL1344 Δ*sopB*Δ*sopE2*::*tet*	Cooper et al 2011[15]
STm *PprgH-gfp*	SB300 *prgh-gfp*	Hockenberry et al 2021[16]
STm *PprgH-gfp* + mCherry	SB300 *prgh-gfp* containing pFPV-*mCherry*	This study
14028 Δ*STM4351*	14028 *STM4351::kan*	Porwollik et al 2014[17]
Δ*STM4351*	IR715 *STM4351::kan*	This study
Candida albicans strains		
C. albicans ATCC	*Candida albicans* (Robin) Berkhout, ATCC[a] #90028	ATCC
C. albicans 529L		Rahman et al. 2007[18]
C. albicans SC5314	wild-type	Gillium et al. 1984[19]
C. albicans (fluorescence microscopy)	SC5314 GFPy	
SC5314 *arg4*Δ/Δ	*arg4*Δ::FRT+/*arg4*Δ::FRT+	This study
SC5314 *arg4*Δ/Δ+*ARG4*	*arg4*Δ::FRT+/*arg4*Δ::FRT+ *NEUT5L/neut5l*Δ::*ARG4*	This study
SC5314 empty vector	*RPS1*/*RPS1*::*SAT1*-tetTA-Pr*tetO*-t*ADH1*	This study
SC5314 *tetO-sopB*	*RPS1*/*RPS1*::*SAT1*-tetTA-Pr*tetO-sopB*-t*ADH1*	This study

Table S15. Plasmids used in this study.

**Plasmid name**	**Relevant feature**	**Source**
pFPV-*mCherry*	ColE1 *ori*, P*rpsM.mCherry*	Drecktrah et al. 2008[20]
SAT1-flipper	FRT-Pr*CaMAL2-FLP-SAT1*-FRT	Reuss et al. 2004[21]
CaHygB-flipper	FRT-Pr*CaMAL2-FLP*-*CaHygB*-FRT	Liu et al. 2022[2]
CIpSATtetTrans	*RPS1-tetR-ScHAP4AD*-Pr*tetO-SAT1*	Steve Saville
CIpSATtetTransMCStADH1	MCS-tADH1 in CIpSATtetTrans	This study

Plasmid name	Relevant feature	Source
CIpSATtetTransMCStADH1-SopB	*Salmonella sopB* in CIpSATtetTransMCStADH1	This study

Table S16. Primers used in this study.

Primer Name	Sequences (5'-3')
Primers used for Sequencing	
ITS1F	CTTGGTCATTTAGAGGAAGTAA
ITS1R	GCTGCGTTCTTCATCGATGC
27F	AGAGTTTGATCMTGGCTCAG
534R	ATTACCGCGGCTGCTGG
Primers targeting Salmonella genes (RT-PCR)	
hilAF	CCGCTGTATTTATGCCTTACG
hilAR	TGCAGACTCTCGGATTGAACC
arcAF	ATAACGGCGTCTCAATTAACCC
arcAR	TGGCGTGGTCGTAATTAATGT C
argFF	GACCGATGAATTTCACCCCAC
argFR	GATATCCATGCCCATCTTCGC
STM4351F	CACGCGTAAAGGGGCTTATC
STM4351R	CAAGACGGTTGTTTTTCAGATCG
gapAF	TTTAGCAGCGCCAGTAGAGG
gapAR	TCGGCATCATCGAAGGTCTG
invAF	ACAGTGCTCGTTTACGACCTGAAT
invAR	ACACCAATATCGCCAGTACG
Primers targeting C. albicans genes (RT-PCR)	
Arg1F	TCCAGTAGAATTATTCATTGAAGCTAA
Arg1R	ACAACCTCTGGATTTAATACCAAT
Arg4F	GAACGTCGATTAGGAGAAATC A
Arg4R	CGAAAATTCTCATATCGGTAGCA
CaSopBDETF	GCAAAAGAAGCTCACAGATTCGC
CaSopBDETR	CCGAAGATATTACTTGCGGTCAATAACC
Act1F	TTGGATTCTGGTGATGGTGTTA

Act1R	TCAAGTCTCTACCAGCCAAATC
Primers used for strain confirmation in STm	
STM4351F	ATGATATTCGCGAGTCGGTGG
STM4351R	AAGAATAGCACGGTGACCAC
Primers used for strain construction in C. albicans	
NAT1INTF	CCCAGATGCGAAGTTAAGTGCG
NEUT5LAMPF	GCTGAATCACTTGATAGGATTTAGTTCCATTATGG
NEUT5LAMPR	GGAATTTCTAGTCACTTGACACGACC
tADH1AMPR-SpeI	TCAACTAGTGAAAACTTGAAACTTGAAAACACCG
ARG4CC9KOF	GCCTCGTAGAGAGGTTAATACTTAGATGTATCATATTTAAAGGTTTTCCCAGTCACGACGT
ARG4CC9KOR	TAGACGTGTTGTGTGTTGTGATGGAAGAAGCTTTTATTTAGTGTGGAATTGTGAGCGGAT
ARG4DETF	GCAGGTGATGAGGATATTCATACTGCT
ARG4DETR	GTGGCATCAATGAAGAACCAGTAGAAT
ARG4INTF	CATCCTATGTGGTACTCCGTTTGTTC
ARG4INTR	AGGACTCTCATTAGAGCTCAACAGACT
FLPINTF	CGCGCGTAATACGACTCACT
FLPINTR	CAAGCGCGCAATTAACCCTC
NEUT5homology-pDIS3F	GGAGGCTCCCCAAAGATTTTATCA
NEUT5homology-pDIS3R	CGATTCGTGTTTAAATAATCAATTGAAAAGAAAATAG
PrARG4OLF	*CCTCGAGGTCGACGGTATCG*GTTGCGAAATGTAGCAATCTATGCTT
tARG4OLR	*ATTTGCTTAGCATGCACGCG*CACTAGTATTGGAGTACAAGGTATCTCTGT
Nt5ADH1upUNIVOLR	CGATACCGTCGACCTCGAGG
ADH1tUNIVOLF	CGCGTGCATGCTAAGCAAAT
SynthGene-F	CGCAGTTACGGATCAGTCAC
SynthGene-Rv2	GAATCATCGTAAGCTATCGTAGCG
ADHterm-F-ClaI	TCAATCGATCGCGTGCATGCTAAGCAAATAG
RP10AMPR	CAGGGCCATAAATATGCCGATTCT
Primers targeting Murine genes (RT-PCR)	
Il17aF	GCTCCAGAAGGCCCTCAGA

Il17aR	AGCTTTCCCTCCGCATTGA
Il22aF	GGCCAGCCTTGCAGATAACA
Il22aR	GCTGATGTGACAGGAGCTGA
TnfaF	ATGGCCTCCCTCTCATCAGT
TnfaR	CTTGGTGGTTTGCTACGACG
IfngF	TCAAGTGGCATAGATGTGGAAGAA
IfngR	TGGCTCTGCAGGATTTTCATG
Cxcl1F	TGCACCCAAACCGAAGTCAT
Cxcl1R	TTGTCAGAAGCCAGCGTTCAC
Nos2F	TTGGGTCTTGTTCACTCCACGG
Nos2R	CCTCTTTCAGGTCACTTTGGTAGG
Il1bF	GCAACTGTTCCTGAACTCAACT
Il1bR	ATCTTTTGGGGTCCGTCCAACT
Il10F	ATAACTGCACCCACTTCCCA
Il10R	GGGCATCACTTCTACCAGGT
ActbF	GGCTGTATTCCCCTCCATCG
ActbR	CCAGTTGGTAACAATGCCATGT
Ido1F	CGACAAGGGCTTCTTCCTCGTC
Ido1R	TGGGTCCACAAAGTCACGCATC
Lcn2F	ACATTTGTTCCAAGCTCCAGGGC
Lcn2R	CATGGCGAACTGGTTGTAGTCCG
Reg3gF	ATGGCTCCTATTGCTATGCC
Reg3gR	GATGTCCTGAGGGCCTCTT
S100a8F	TGTCCTCAGTTTGTGCAGAATATAAA
S100a8R	TCACCTCGCAAGGAACTCC
S100a9F	GGTGGAAGCACAGTTGGCA
S100a9R	GTGTCCAGGTCCTCCATGATG
Il1bF	GCAACTGTTCCTGAACTCAACT
Il1bR	ATCTTTTGGGGTCCGTCCAACT
PtgesF	ACGACATGGAGACAATCTATCCT
PtgesR	TGAGGACAACGAGGAAATGT
Ifnl2F	AGCTGCAGGCCTTCAAAAAG

Ifnl2R	TGGGAGTGAATGTGGCTCAG
IfnbF	TCAGAATGAGTGGTGGTTGC
IfnbR	GACCTTTCAAATGCAGTAGATTCA
Il18F	ACTGTACAACCGCAGTAATACGC
Il18R	AGTGAACATTACAGATTTATCCC
TgfbF	CCTGCAAGACCATCGACATG
TgfbR	TGTTGTACAAAGCGAGCACC
Il10F	ATAACTGCACCCACTTCCCA
Il10R	GGGCATCACTTCTACCAGGT
Arg1F	AAGAAAAGGCCGATTCACCT
Arg1R	CACCTCCTCTGCTGTCTTCC
Arg2F	ACAGGGTTGCTGTCAGCTCT
Arg2R	TGATCCAGACAGCCATTTCA

Underlined sequences indicate regions that bind to *SAT1*- and Ca*HygB*-flipper plasmids. Sequences italicized and underlined indicate reverse complementarity to Nt5ADH1upUNIVOL-R and ADH1tUNIVOL-F primers.

Supplementary Figures:

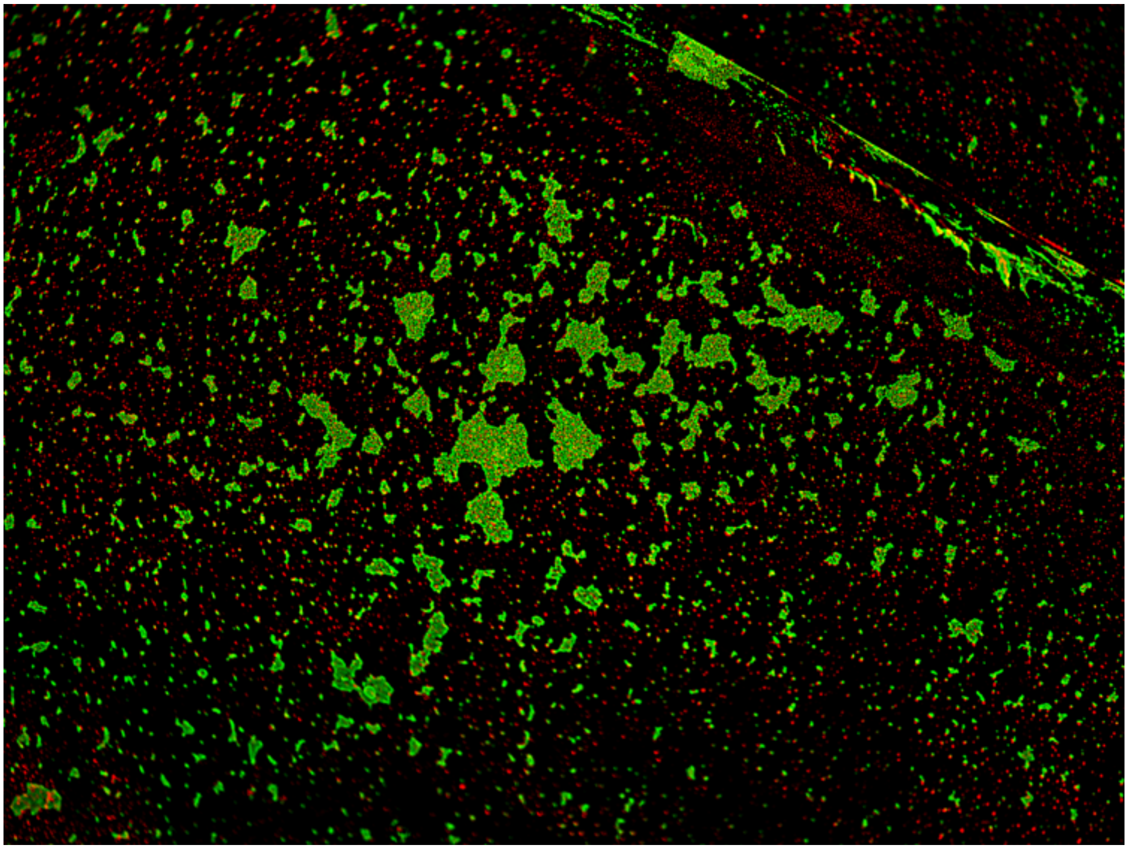

Fig S1. (a) Uncropped Image1 of Fig 2c

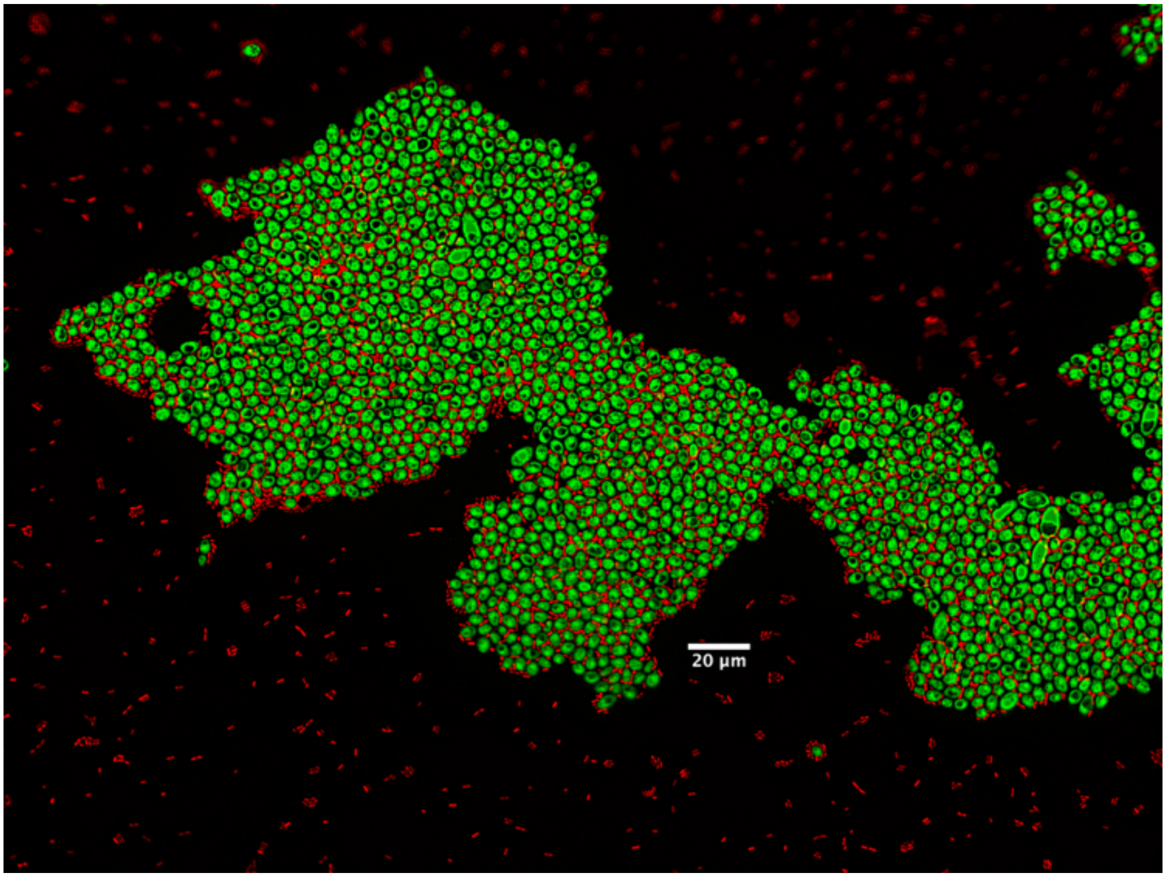

Fig S1. (b) Uncropped Image2 of Fig 2c

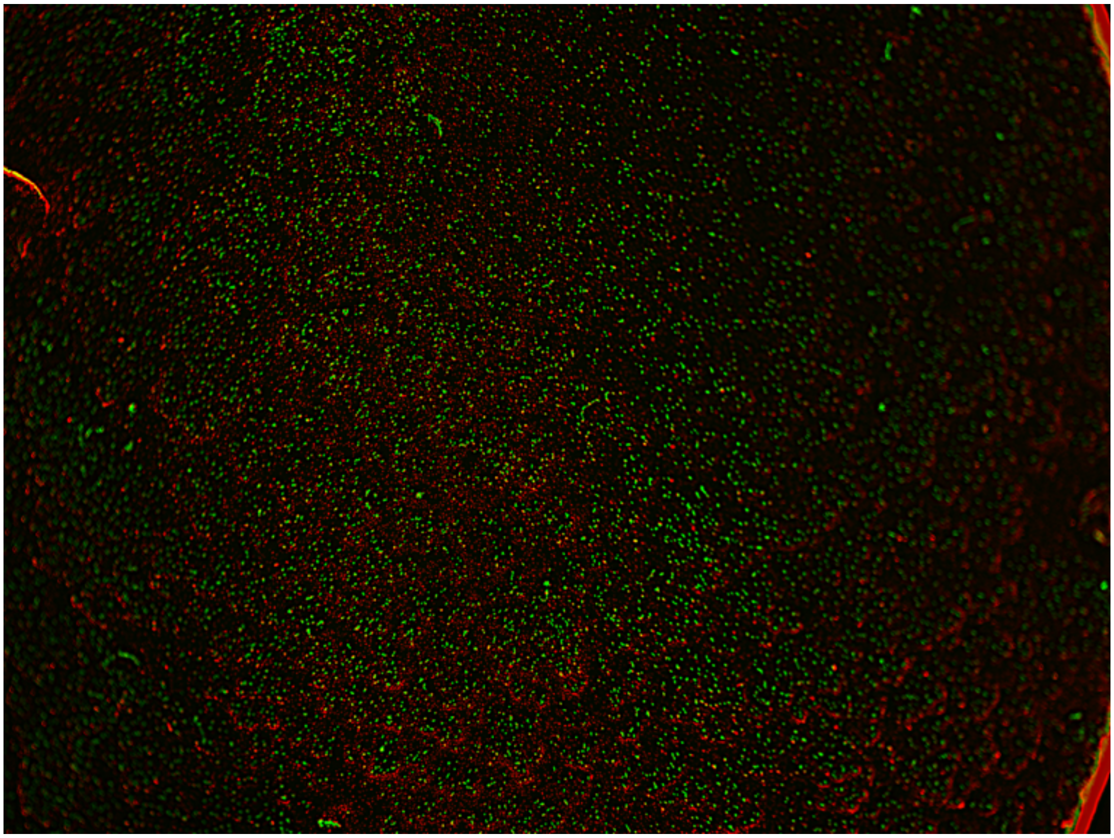

Fig S1. (c) Uncropped Image3 of Fig 2c

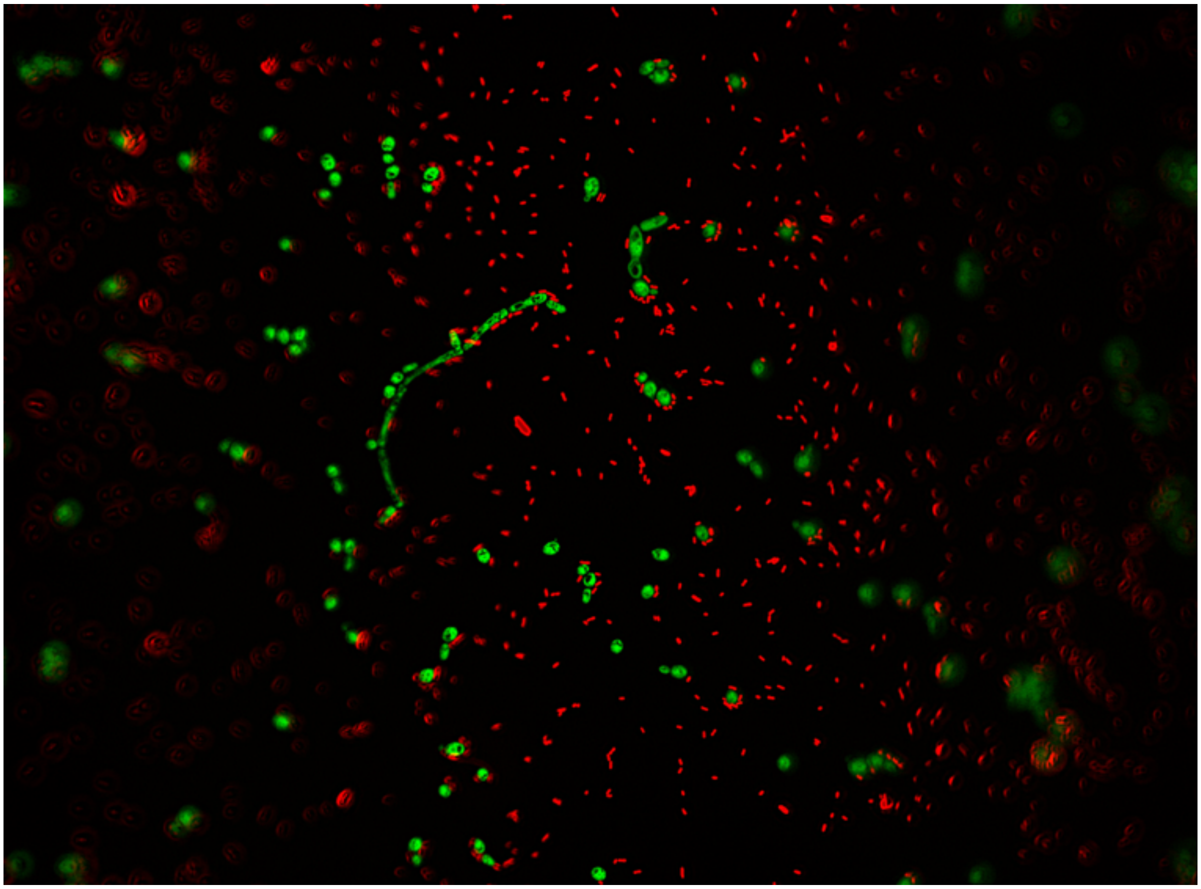

Fig S1. (d) Uncropped Image4 of Fig 2c

Figure S1 a-d. Uncropped Images of Fig. 2c. *C. albicans* green, STm red.

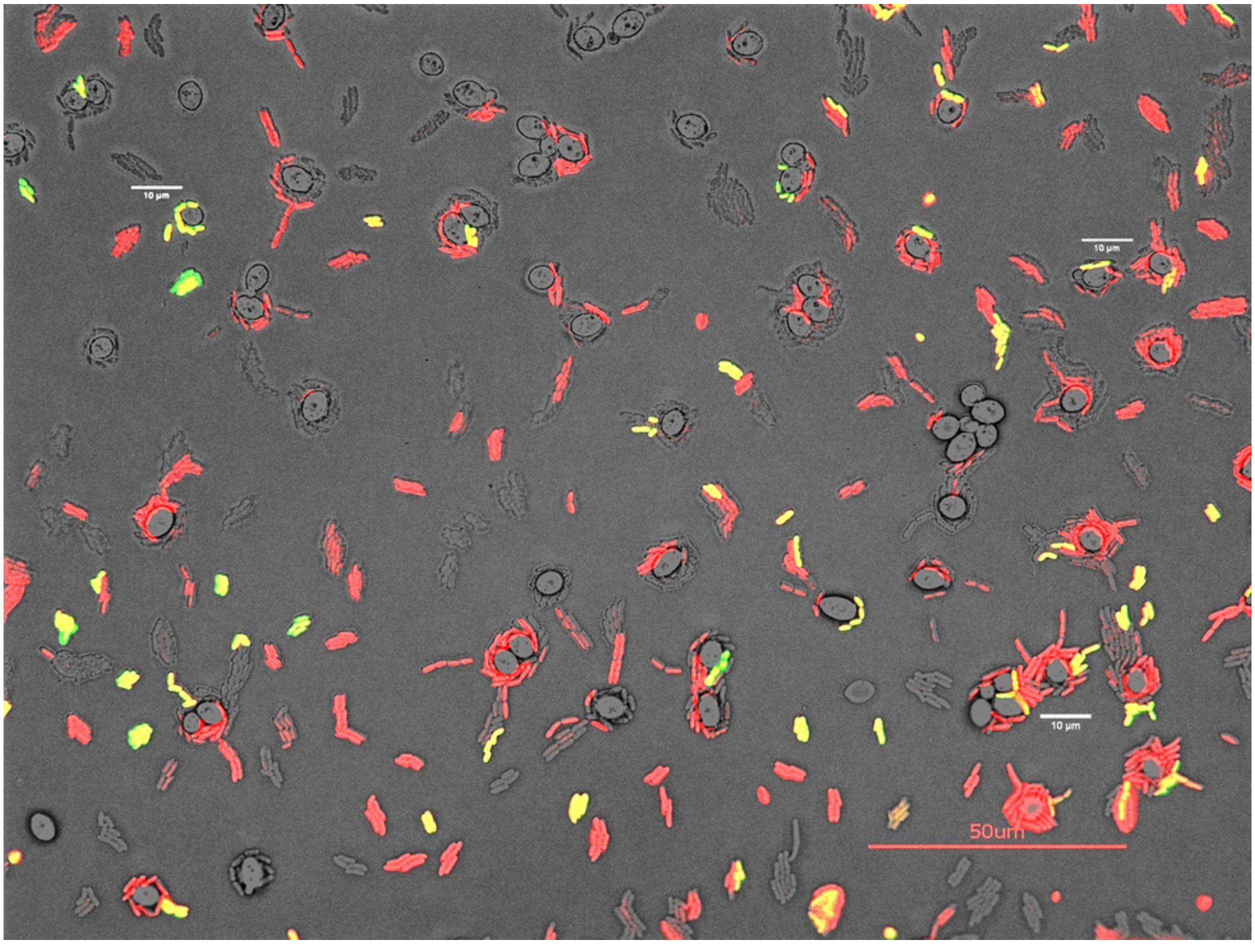

Figure S2. Uncropped Image of Fig. 2f. *C. albicans* brightfield, STm T3SS- red and STm T3SS^{+} green.

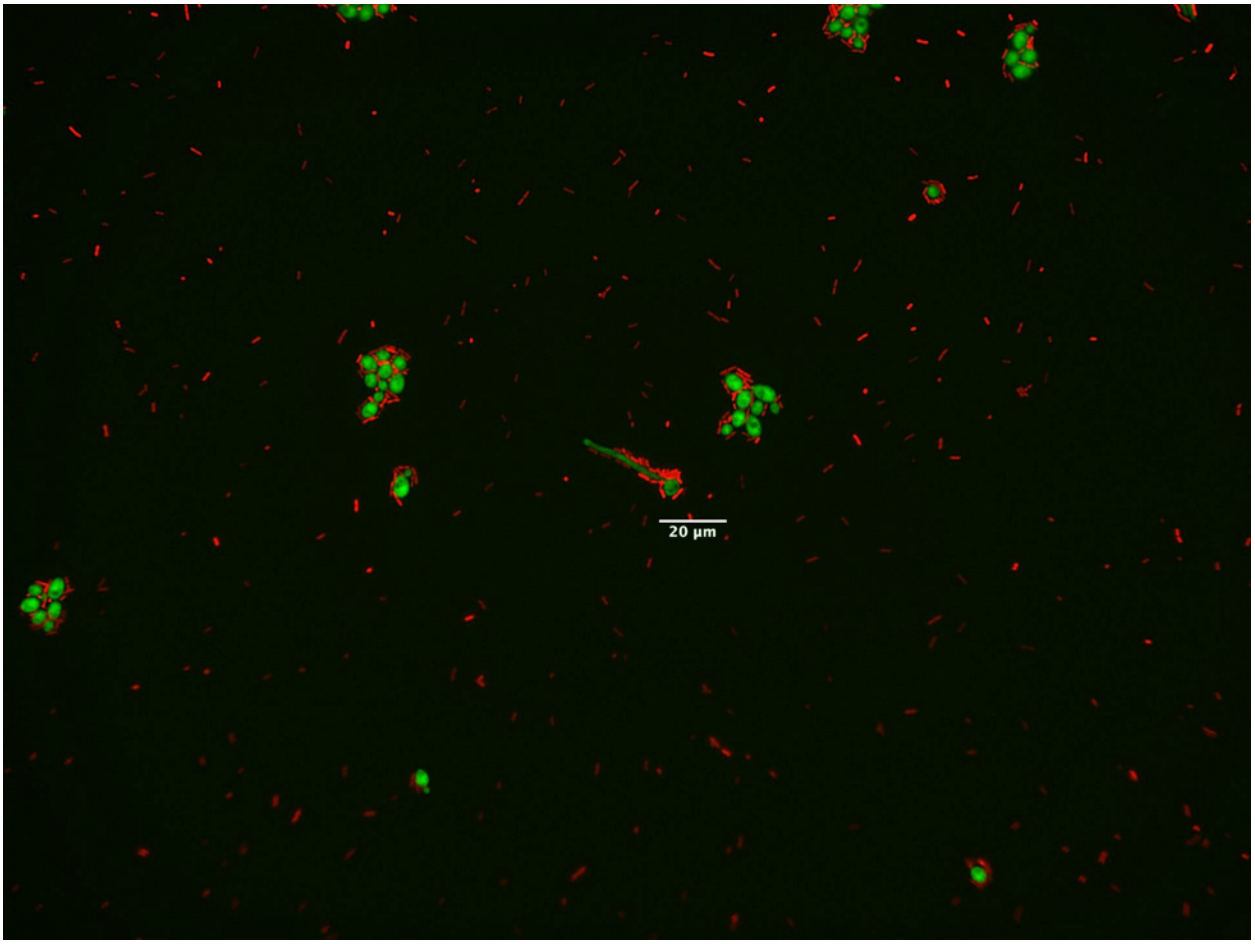

Figure S3. Uncropped Image of Extended Fig. 2f. *C. albicans* green, STm red.

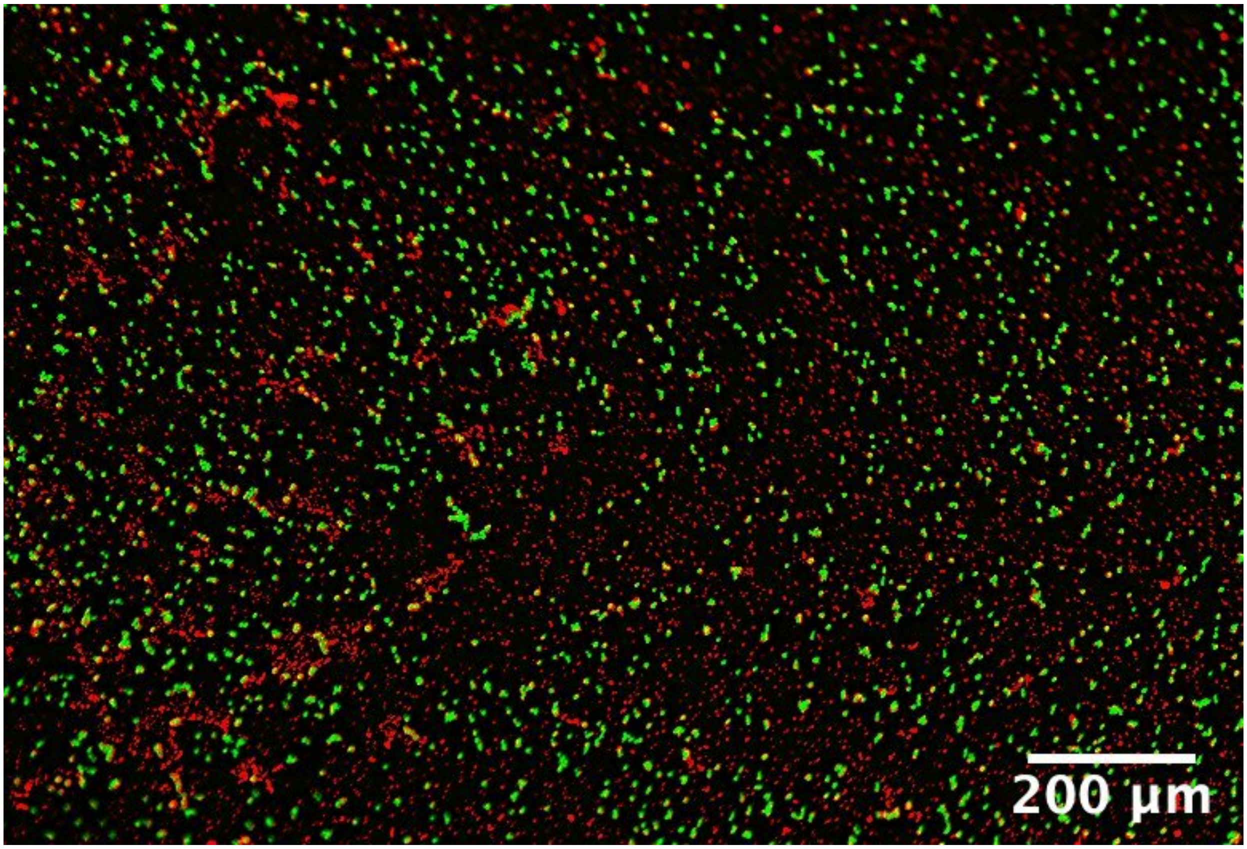

Fig S4. (a) Image 1 Extended Fig. 2g.

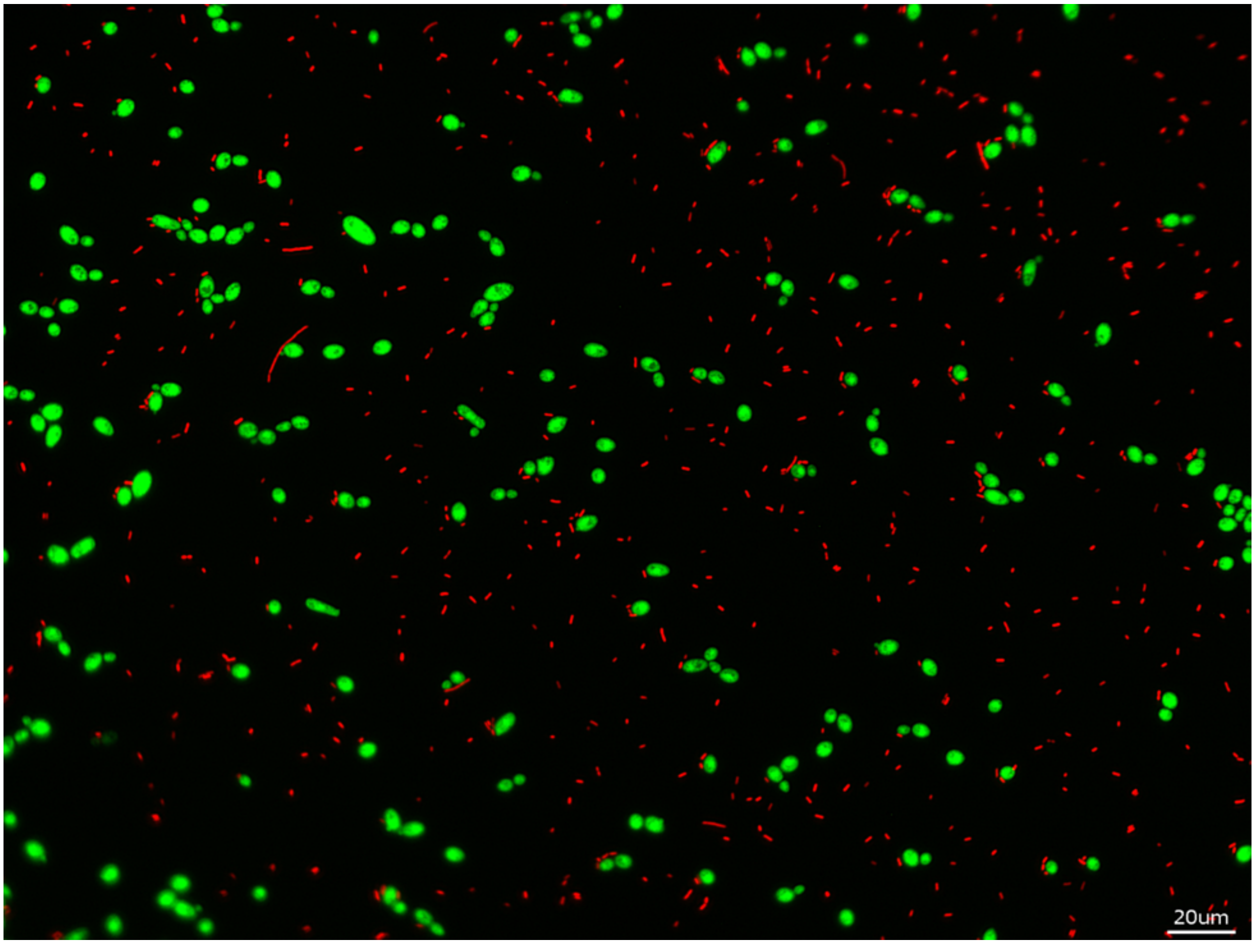

Fig S4. (b) Image 2 Extended Fig. 2g.

Figure S4 a-b. Uncropped Image of Extended Fig. 2g. *C. albicans* green, STm red.

Images used for STm + *C. albicans* cultures:

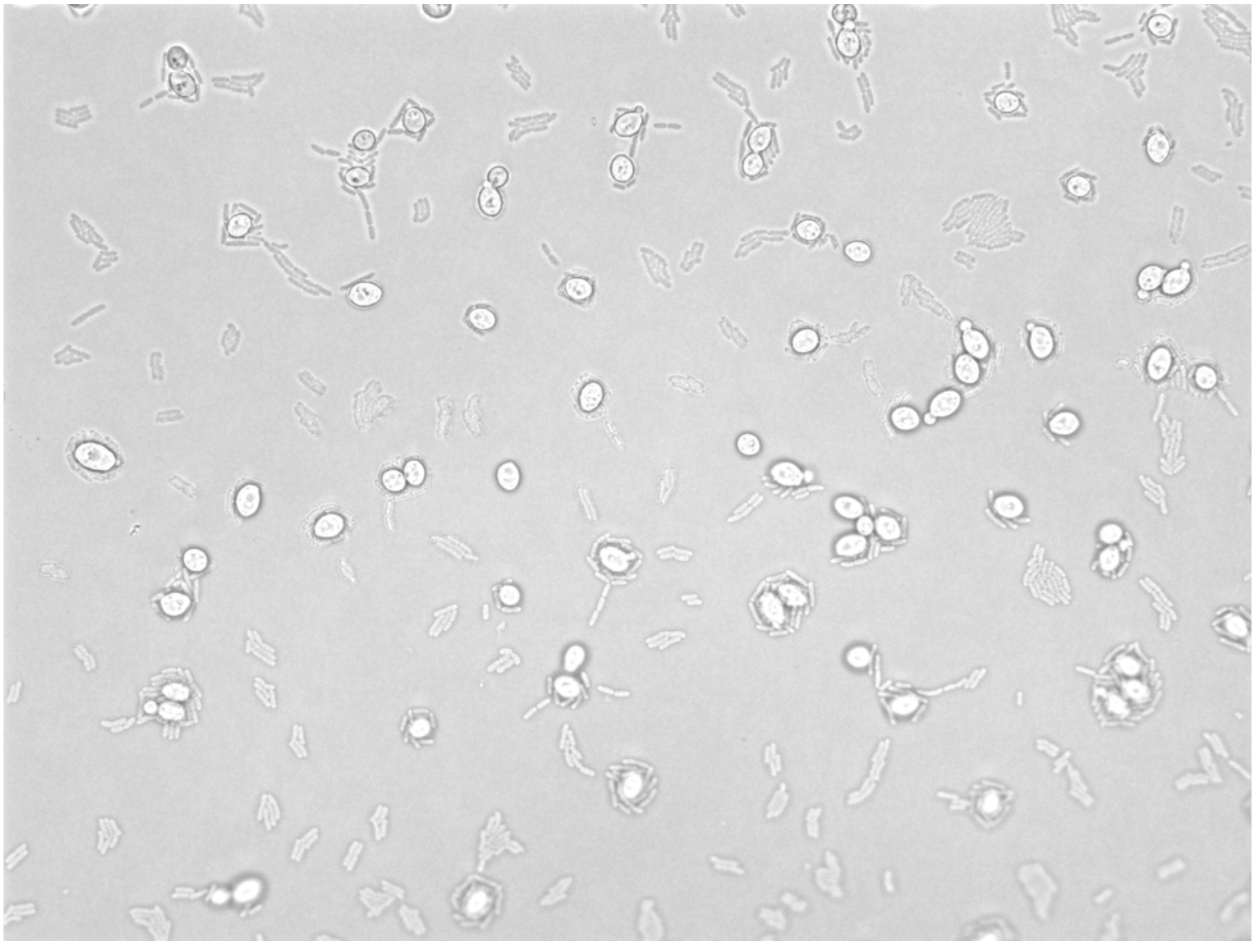

Fig S5. (a) Image1, Brightfield.

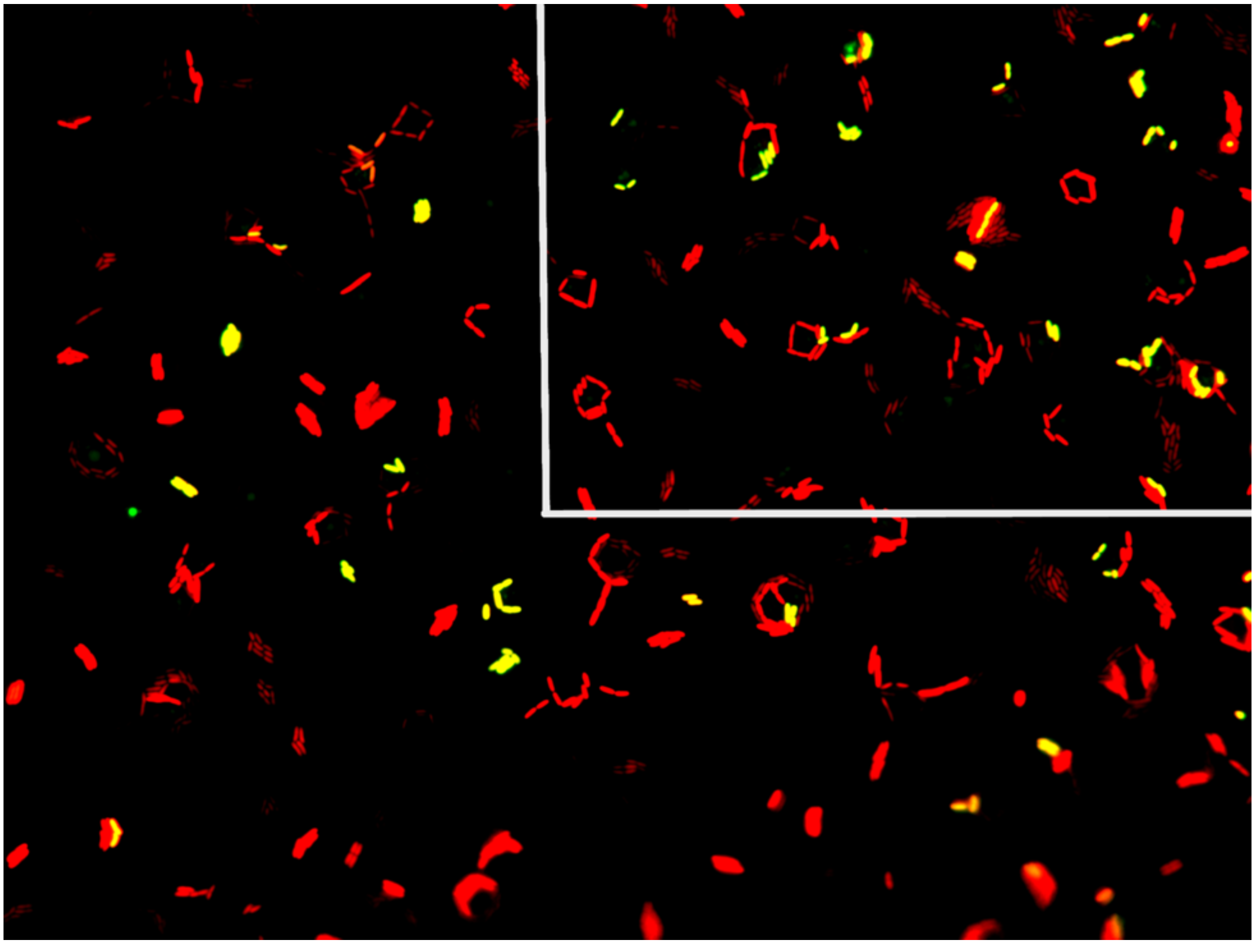

Fig S5. (b) Image1, Fluorescent.

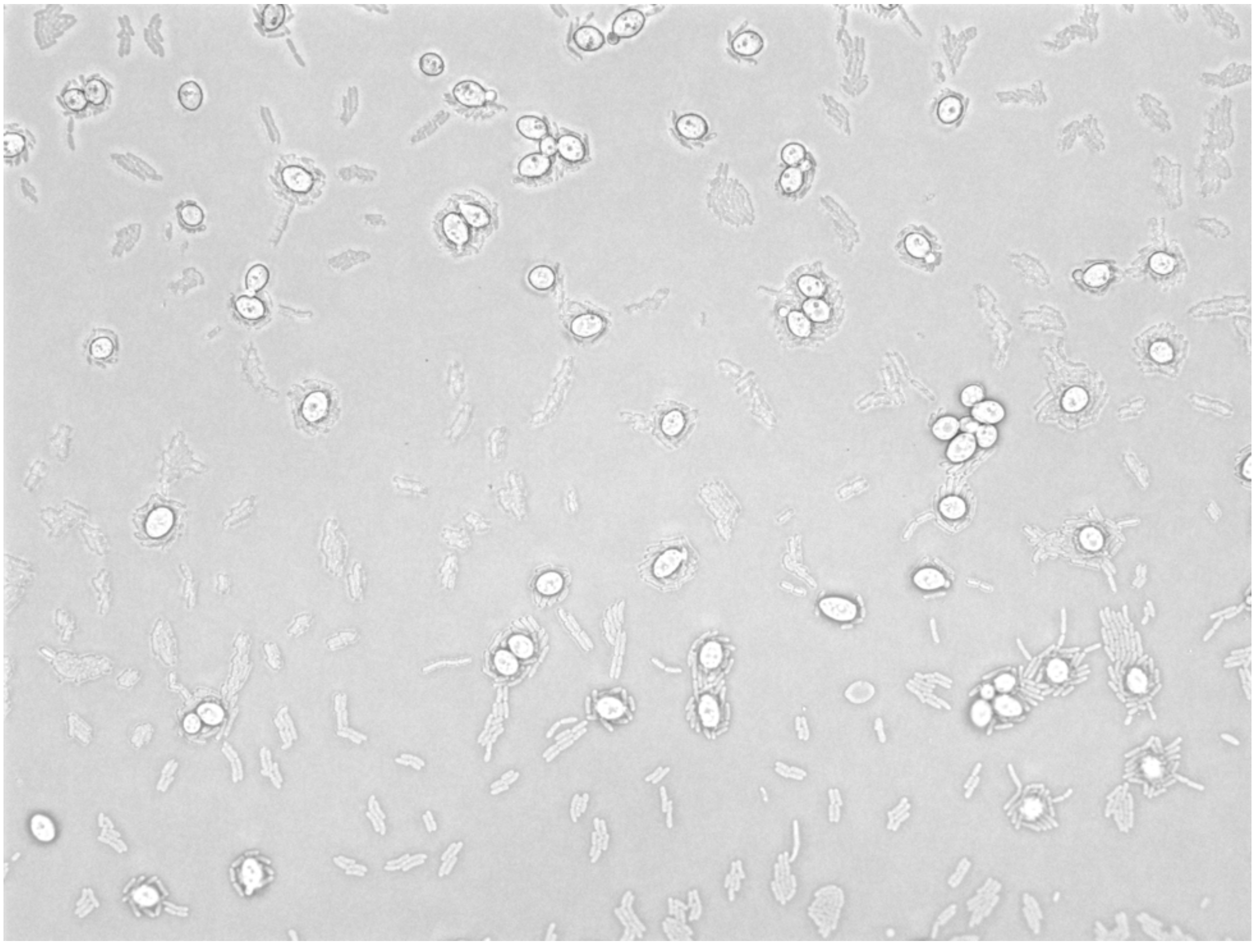

Fig S5. (c) Image2, Brightfield.

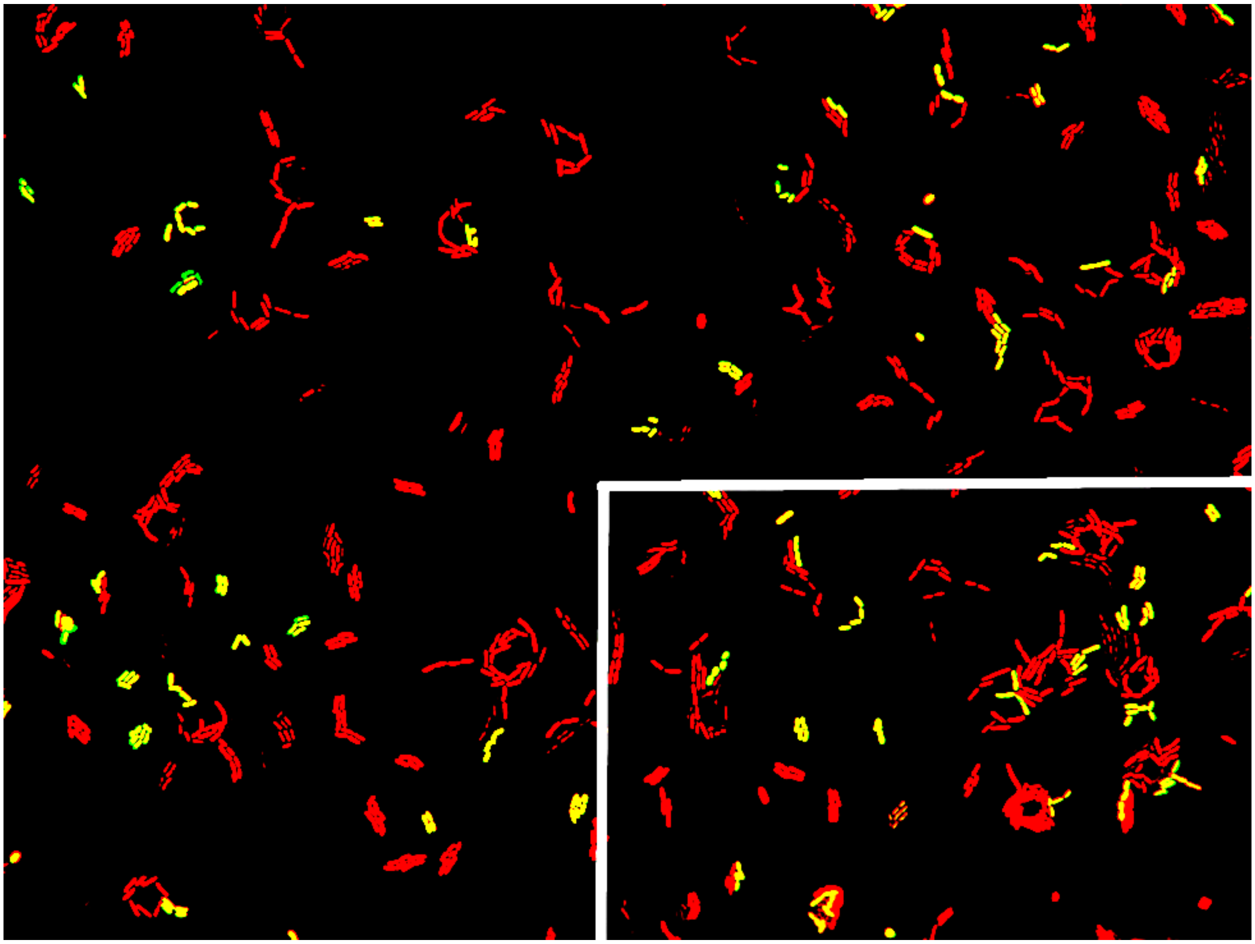

Fig S5. (d) Image2, Fluorescent.

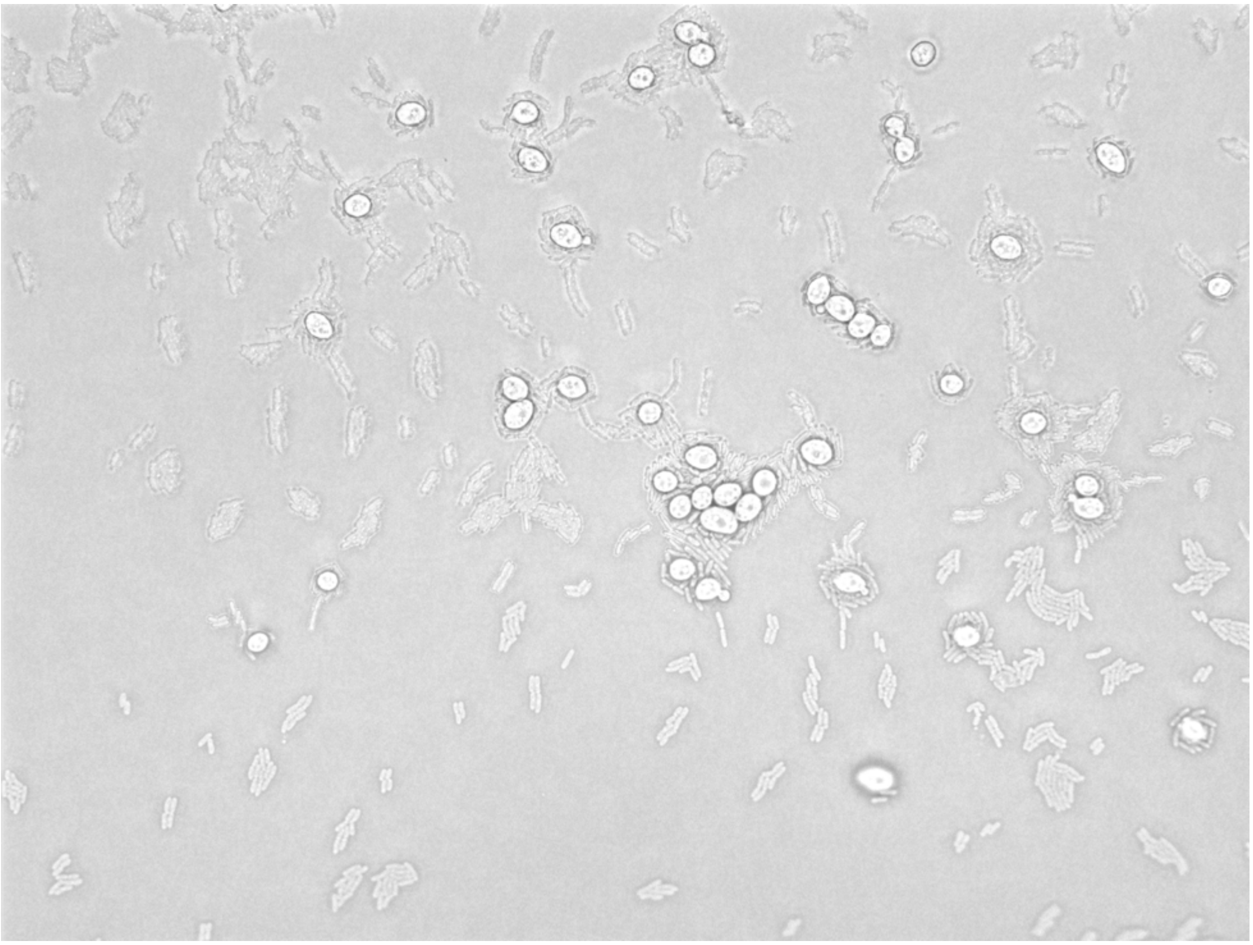

Fig S5. (e) Image3, Brightfield.

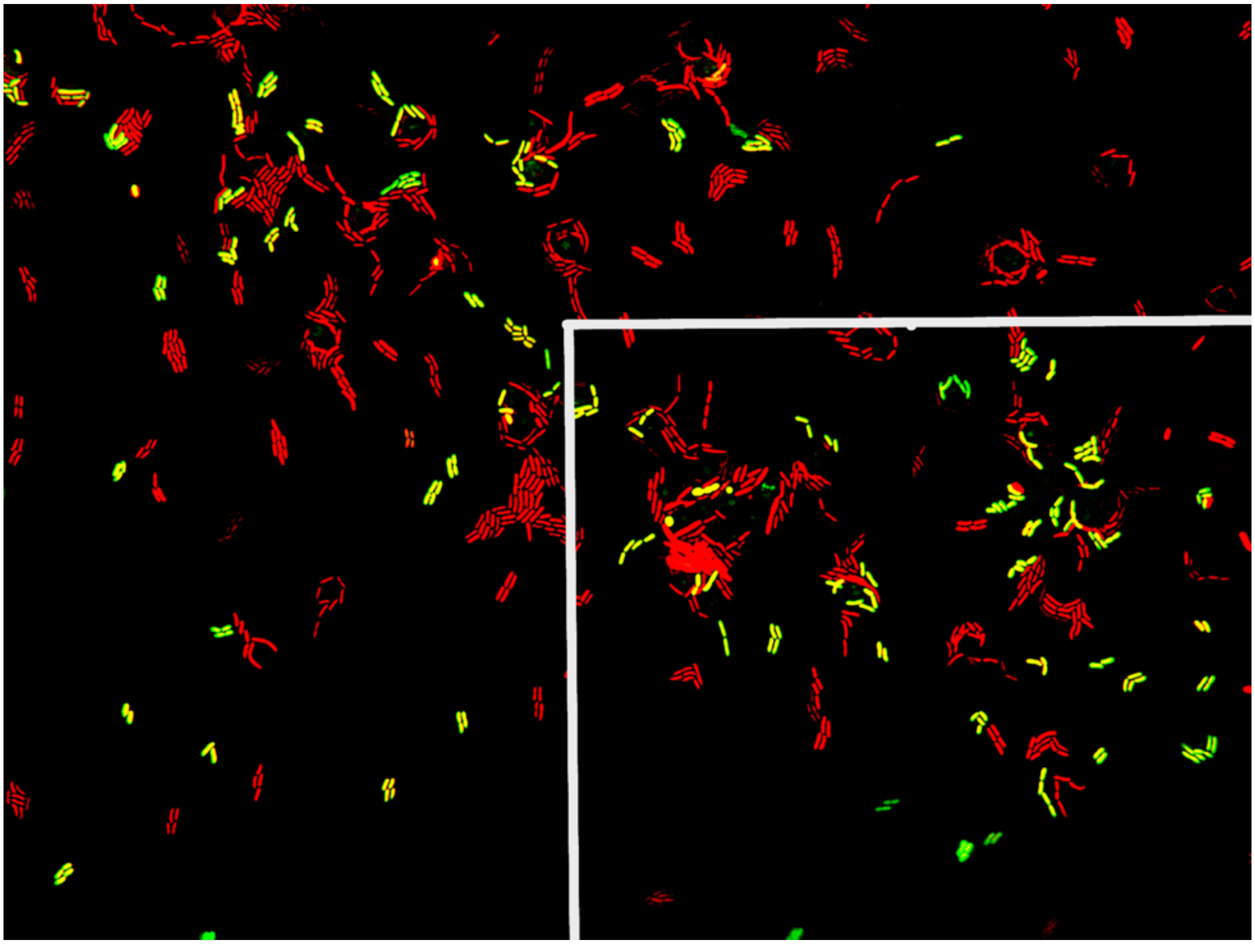

Fig S5. (f) Image3, Fluorescent.

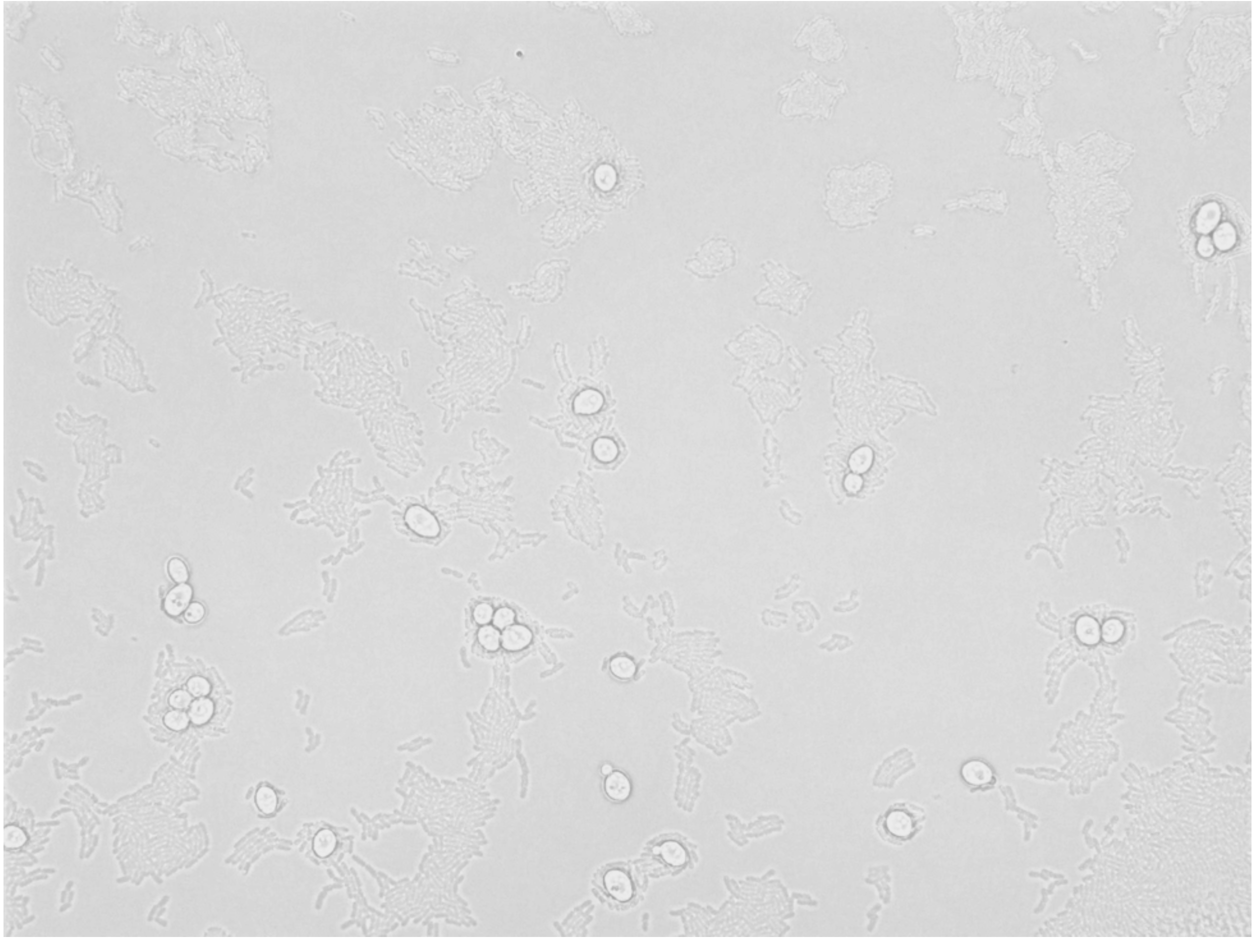

Fig S5. (g) Image4, Brightfield.

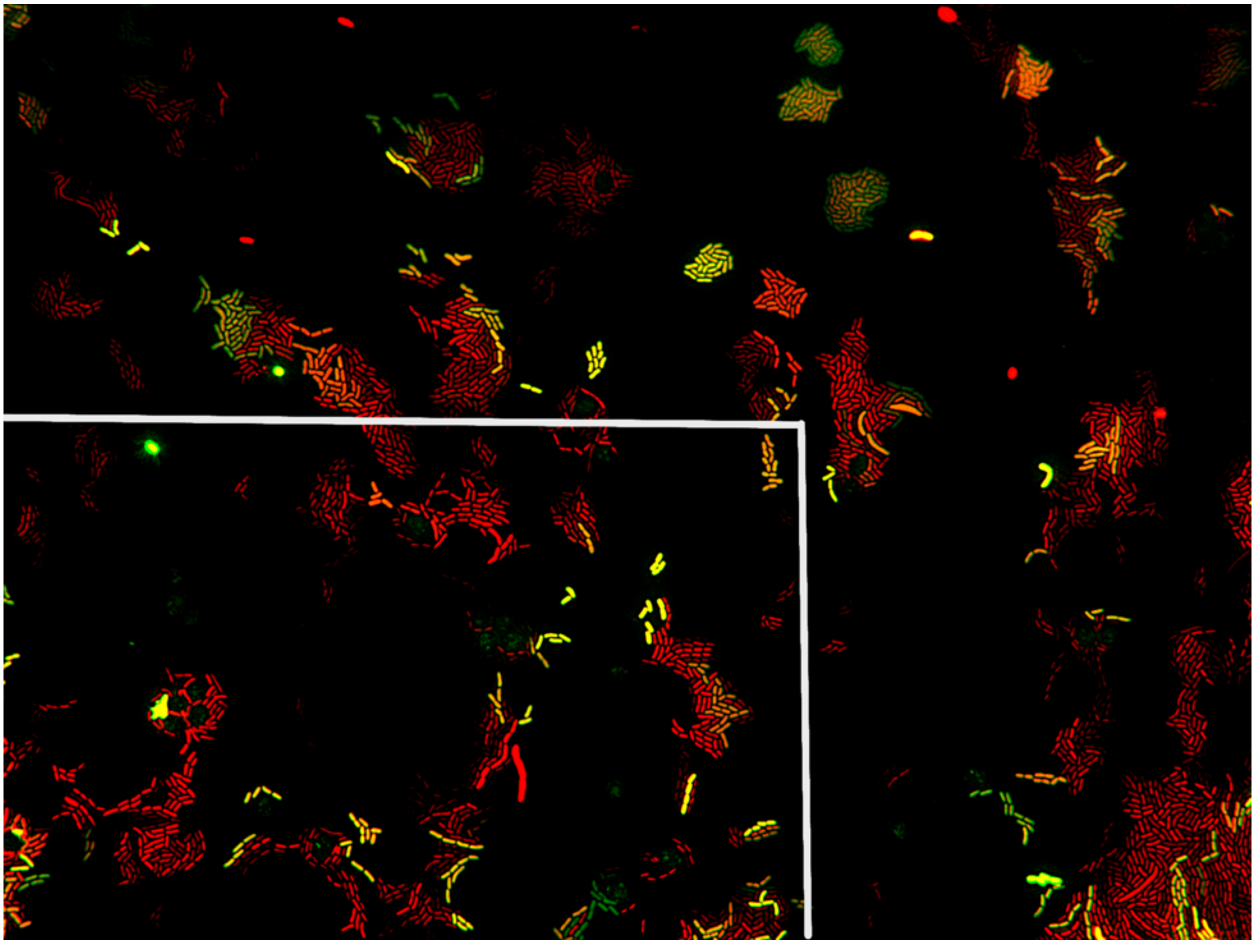

Fig S5. (h) Image4, Fluorescent.

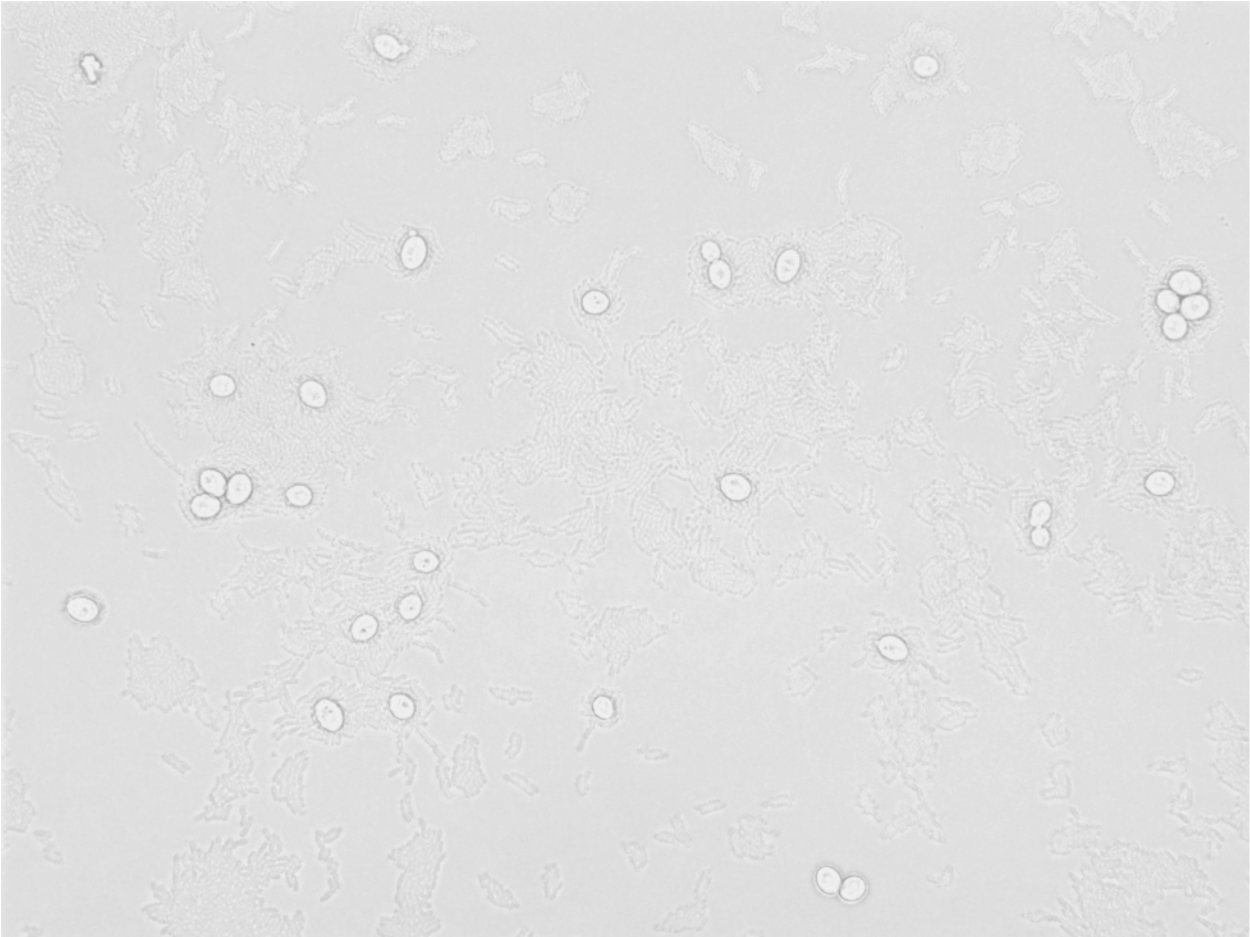

Fig S5. (i) Image5, Brightfield.

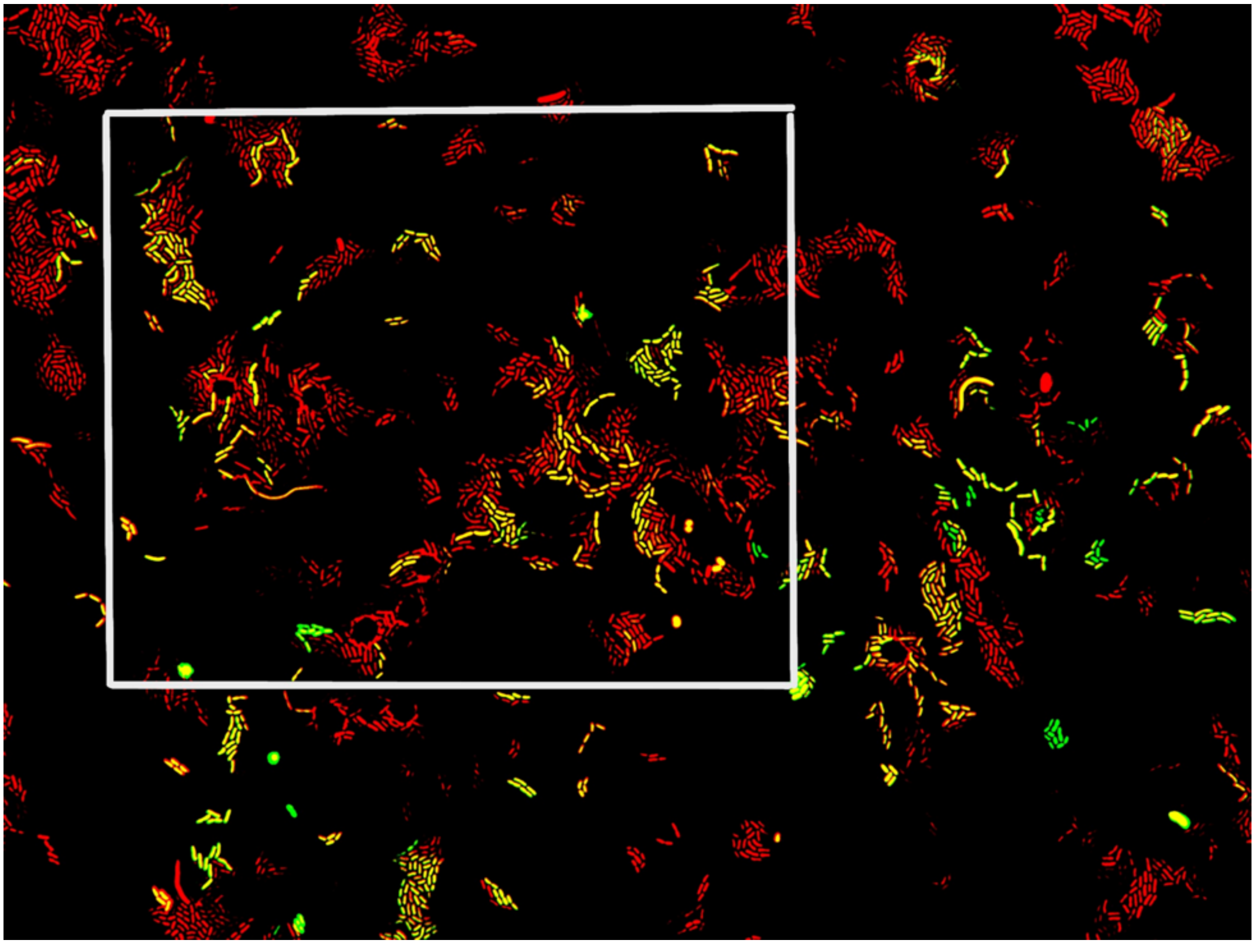

Fig S5. (j) Image5, Fluorescent.

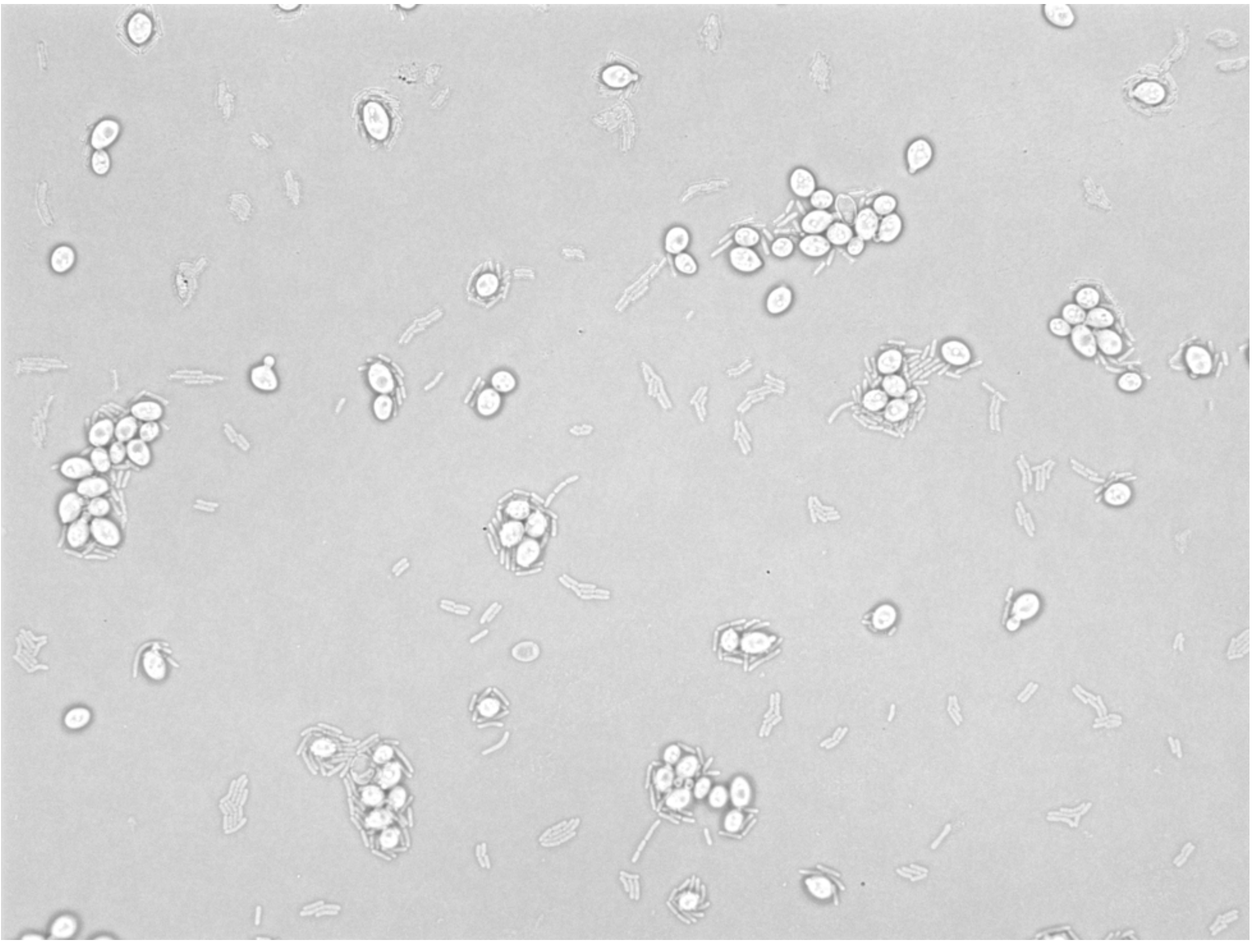

Fig S5. (k) Image6, Brightfield.

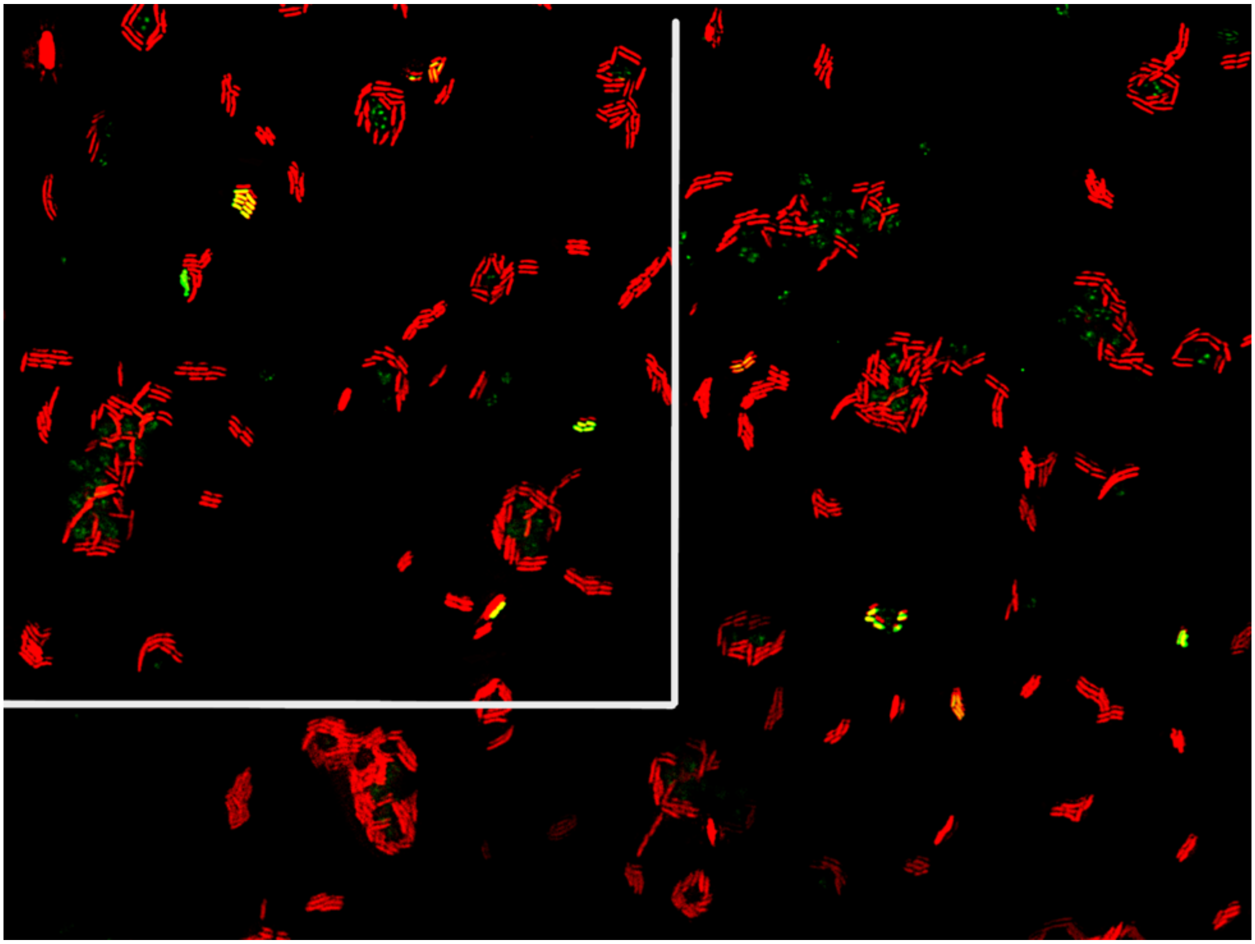

Fig S5. (I) Image6, Fluorescent.

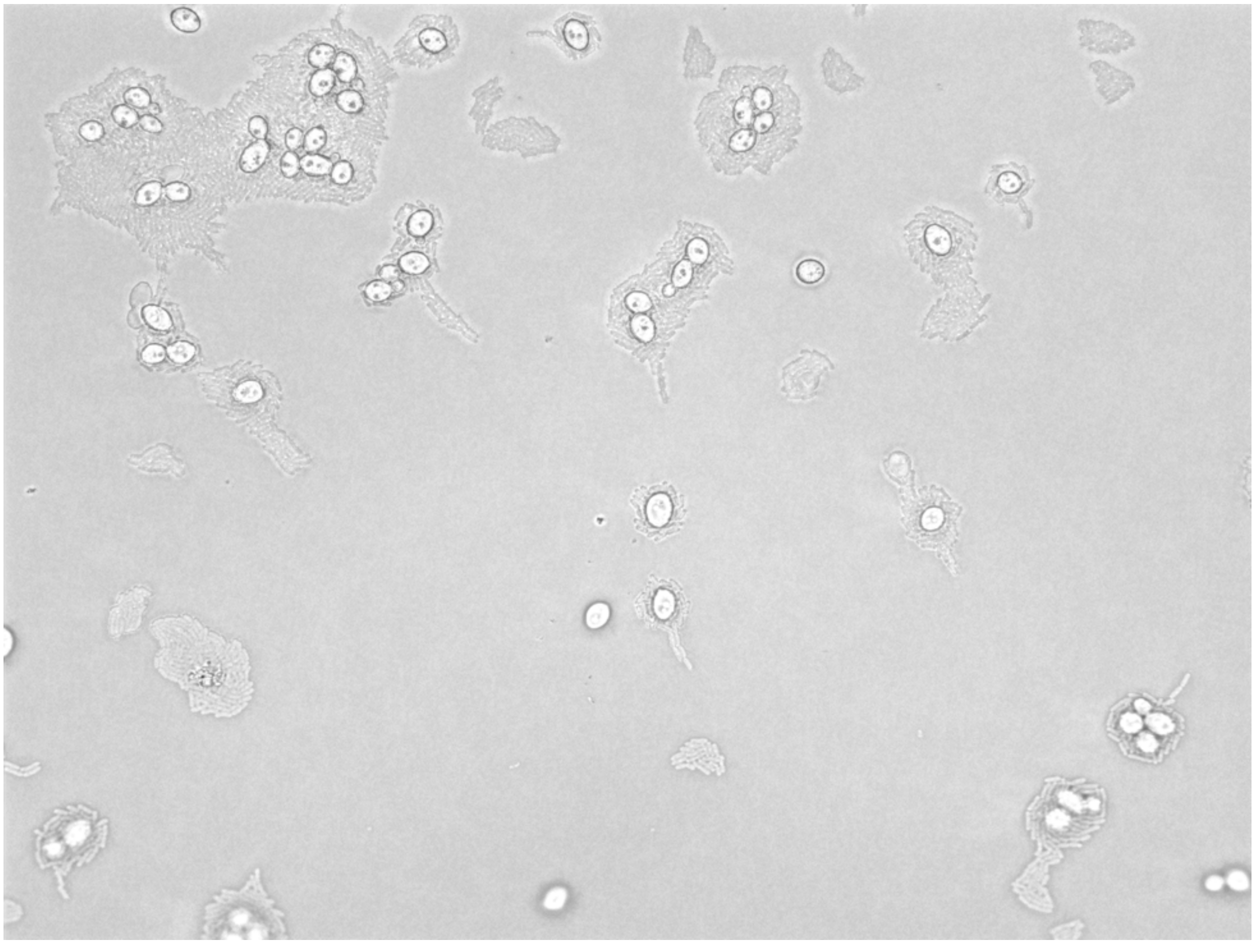

Fig S5. (m) Image7, Brightfield.

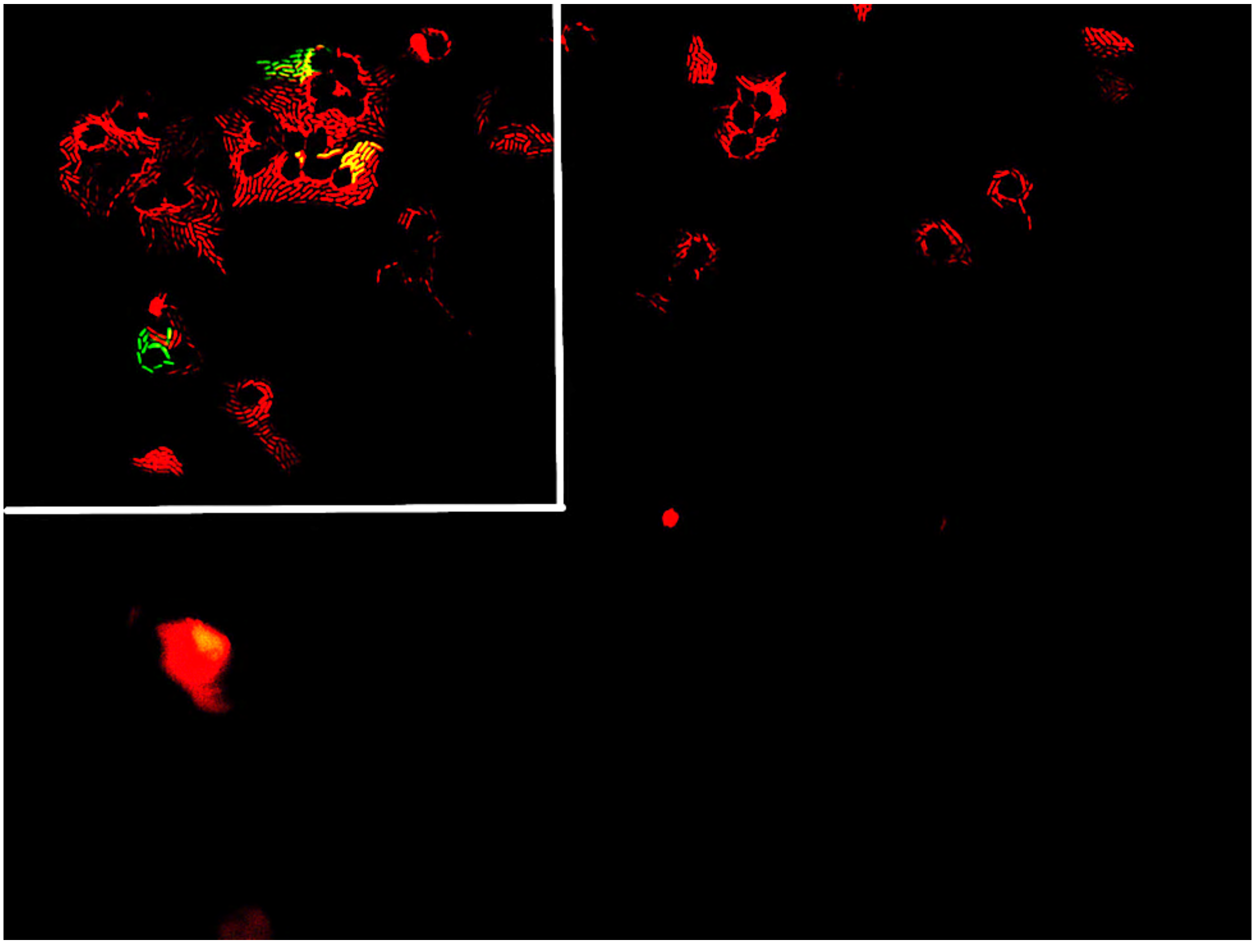

Fig S5. (n) Image7, Fluorescent.

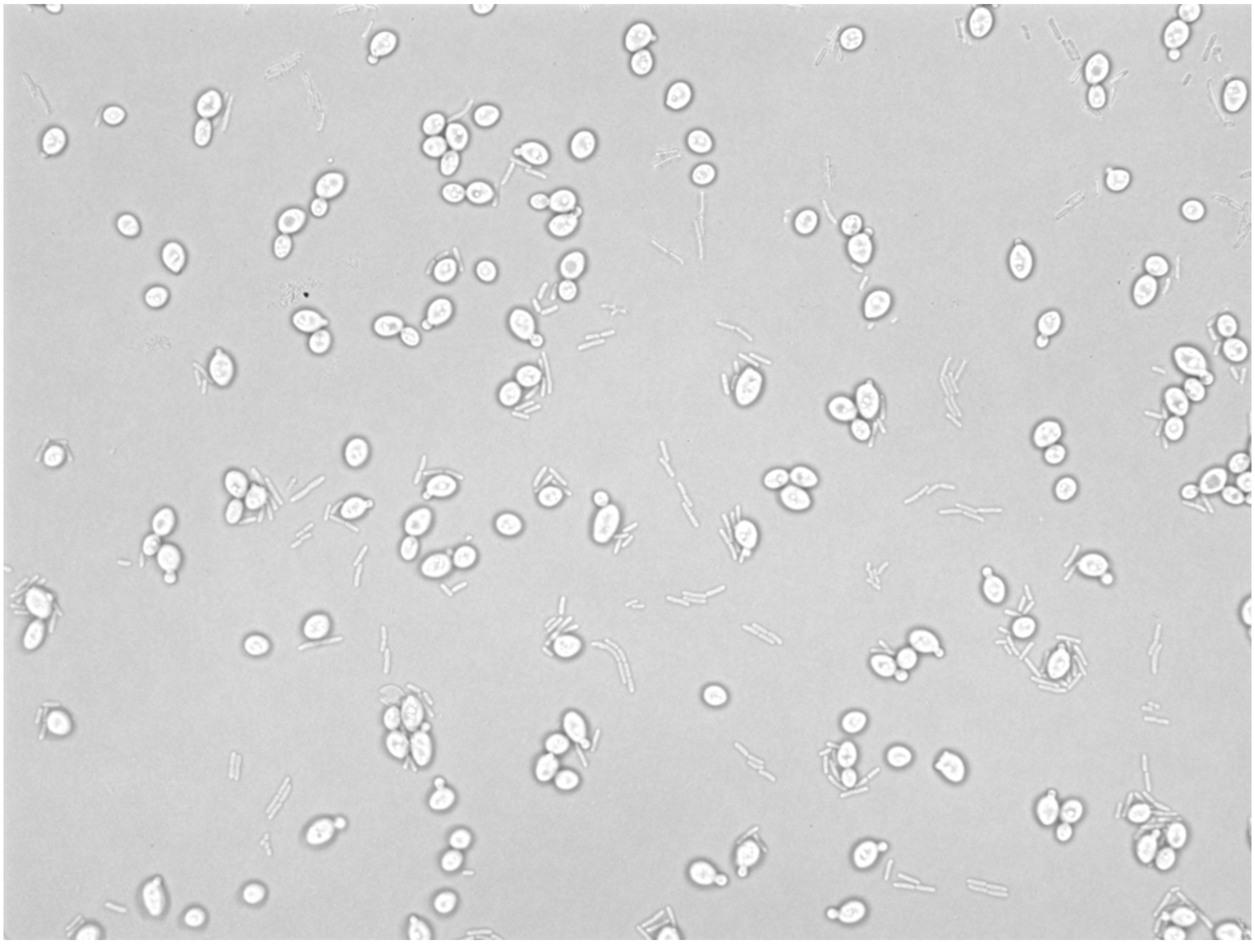

Fig S5. (o) Image8, Brightfield.

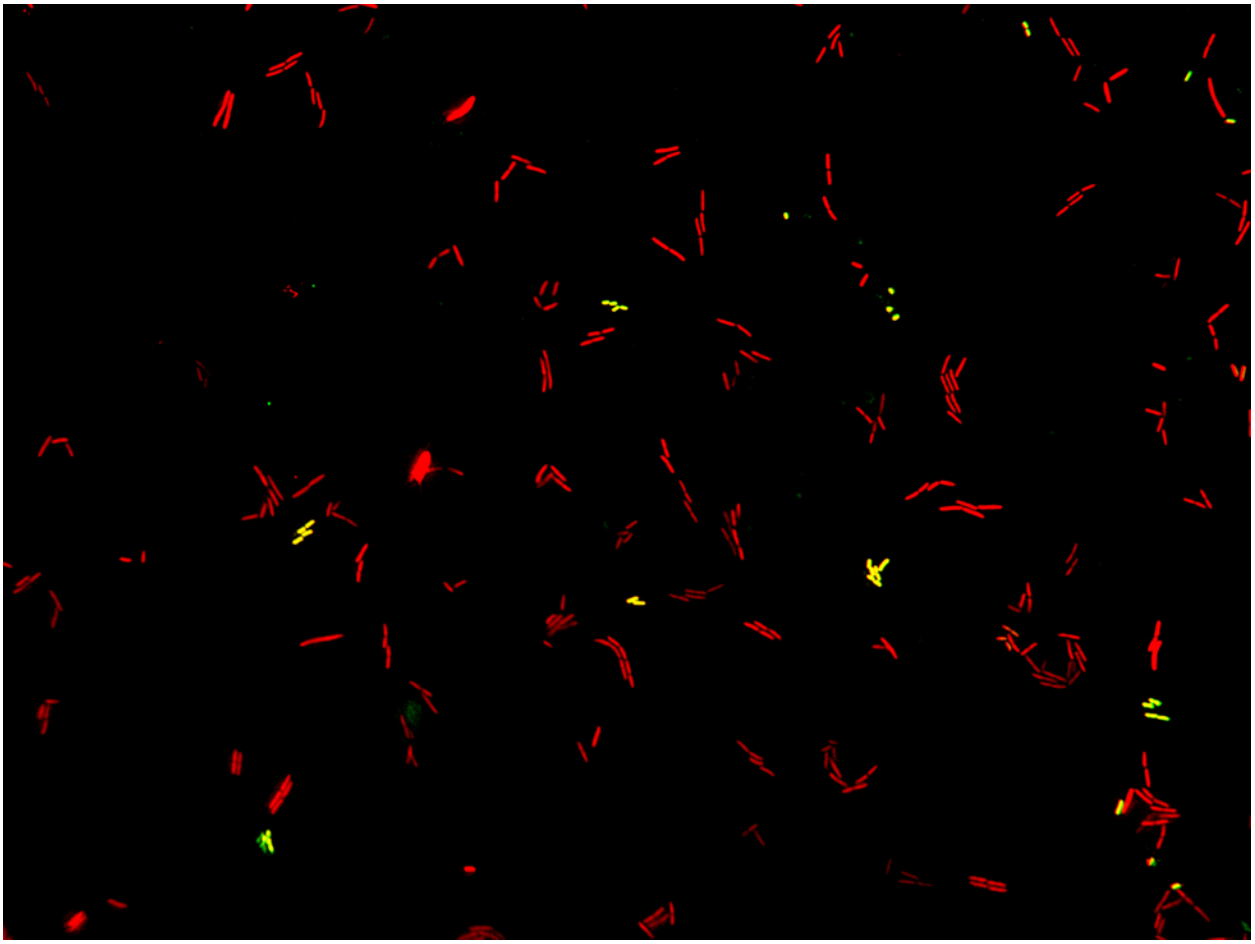

Fig S5. (p) Image8, Fluorescent.

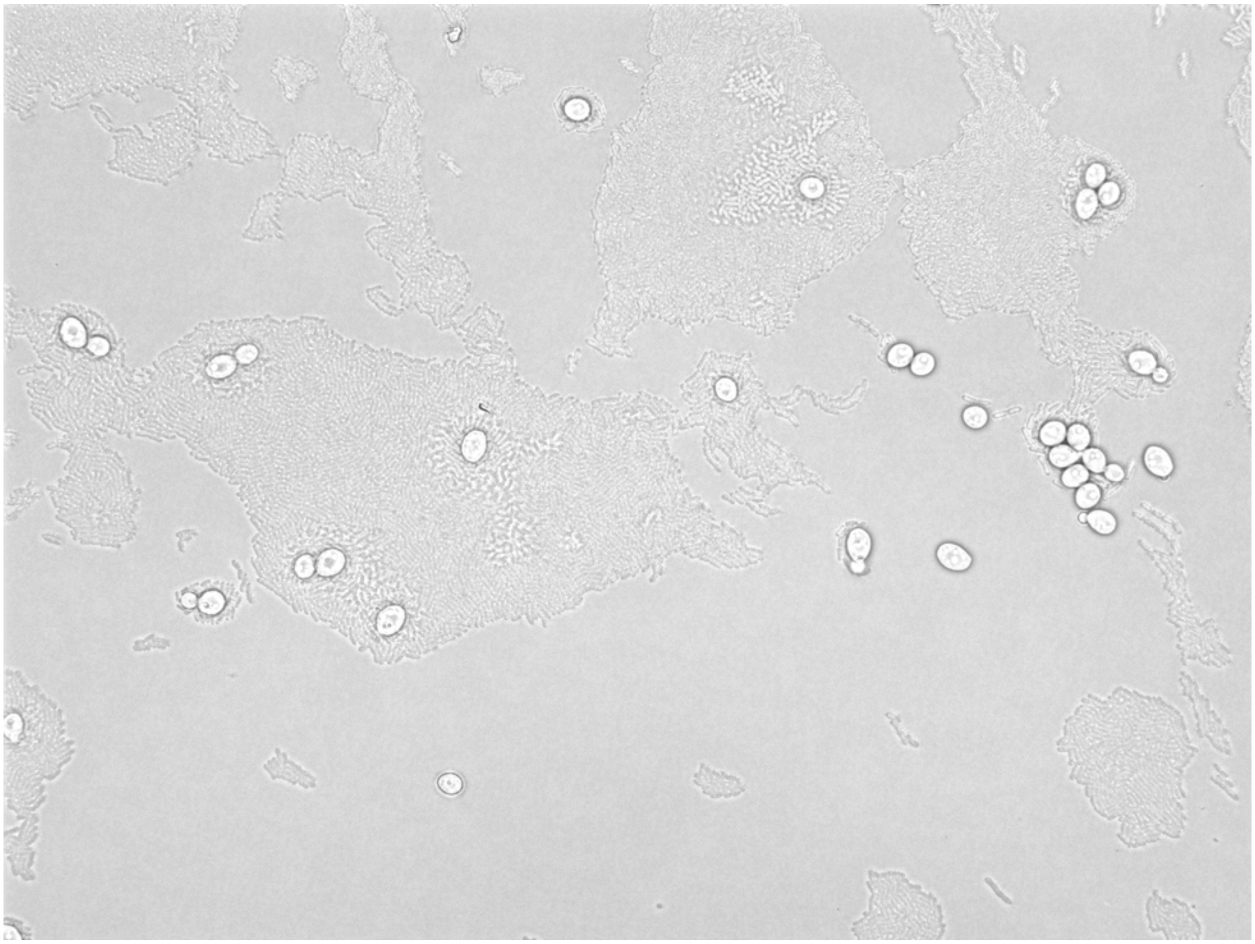

Fig S5. (q) Image9, Brightfield.

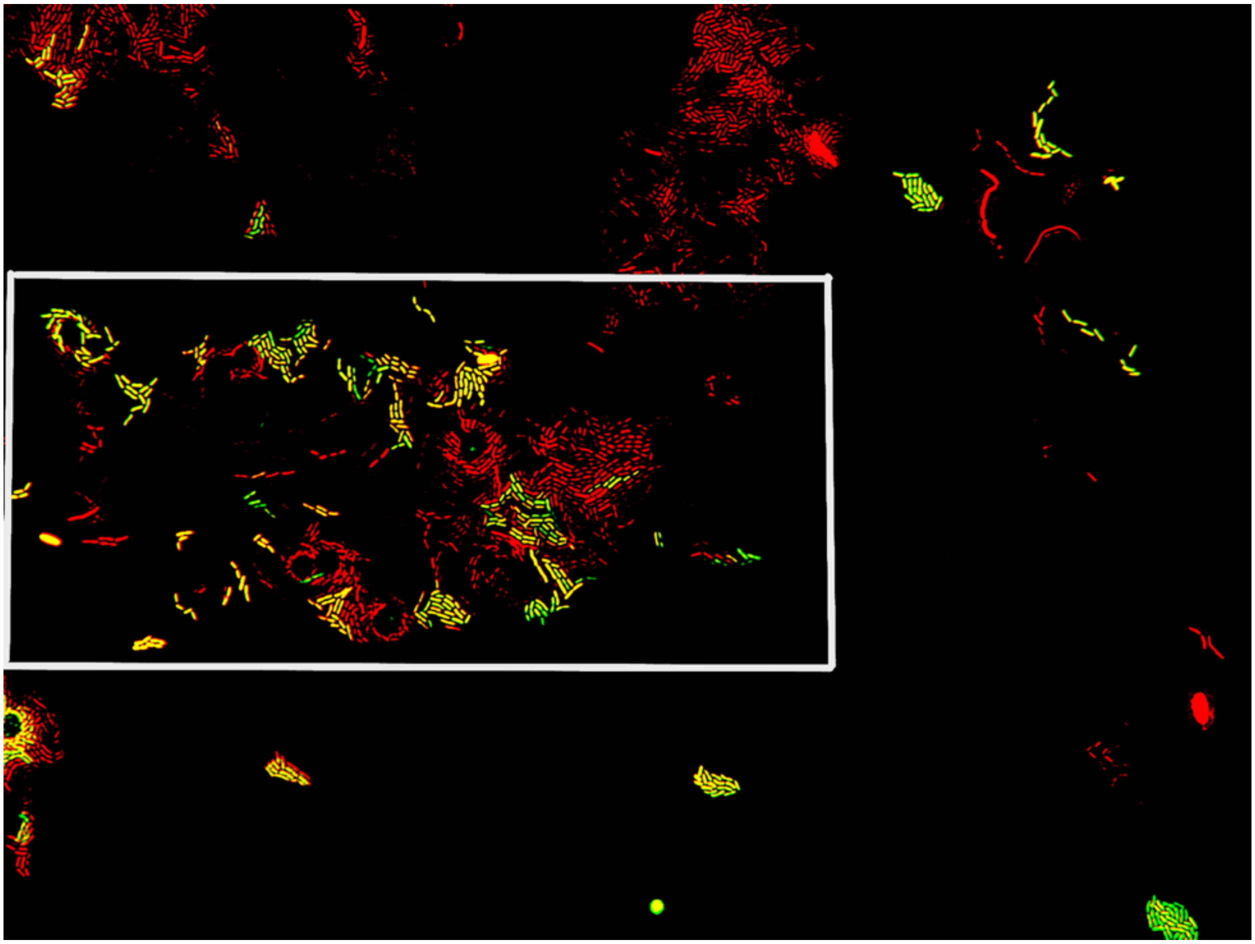

Fig S5. (r) Image9, Fluorescent.

Images used for STm alone:

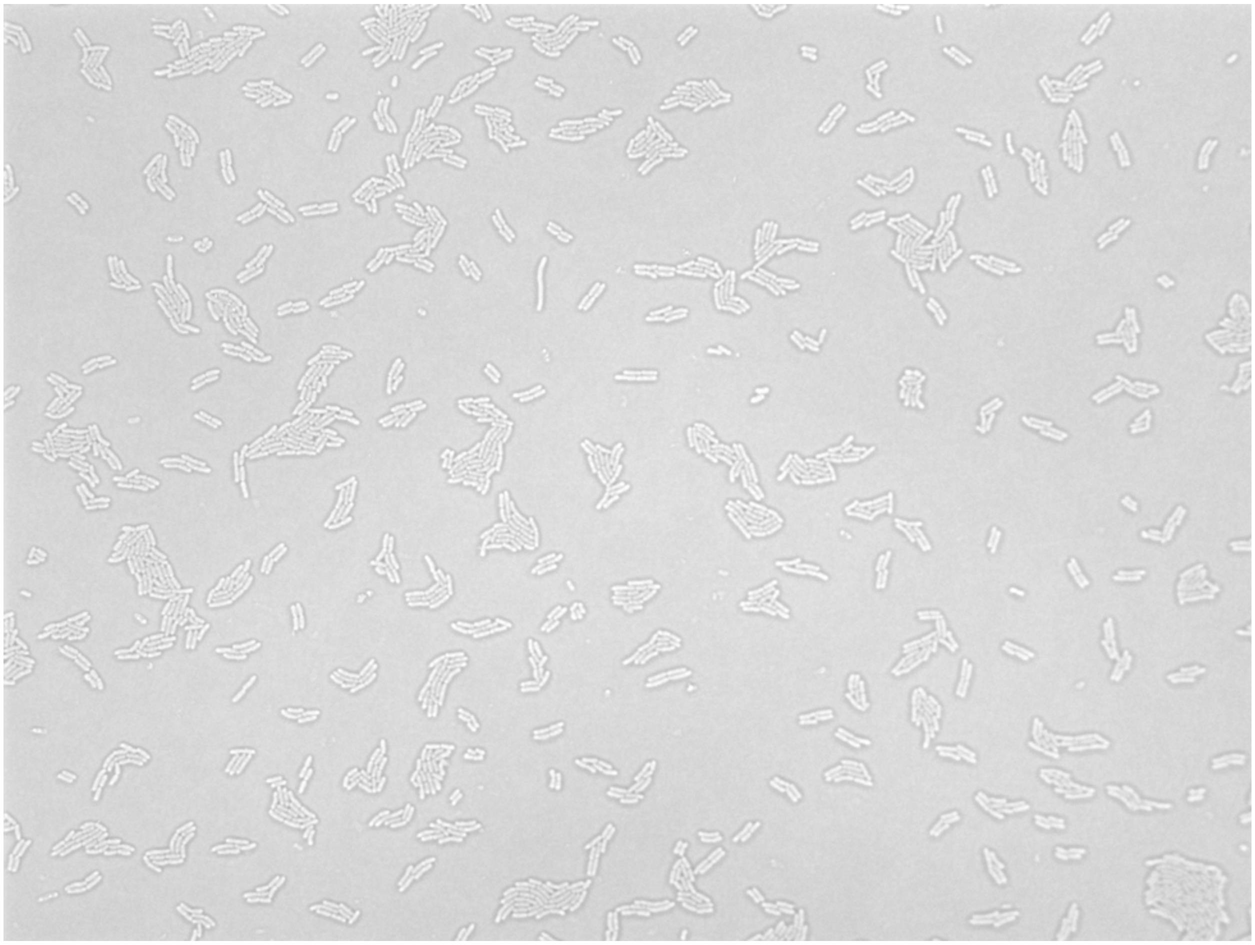

Fig S5. (s) Image1, Brightfield.

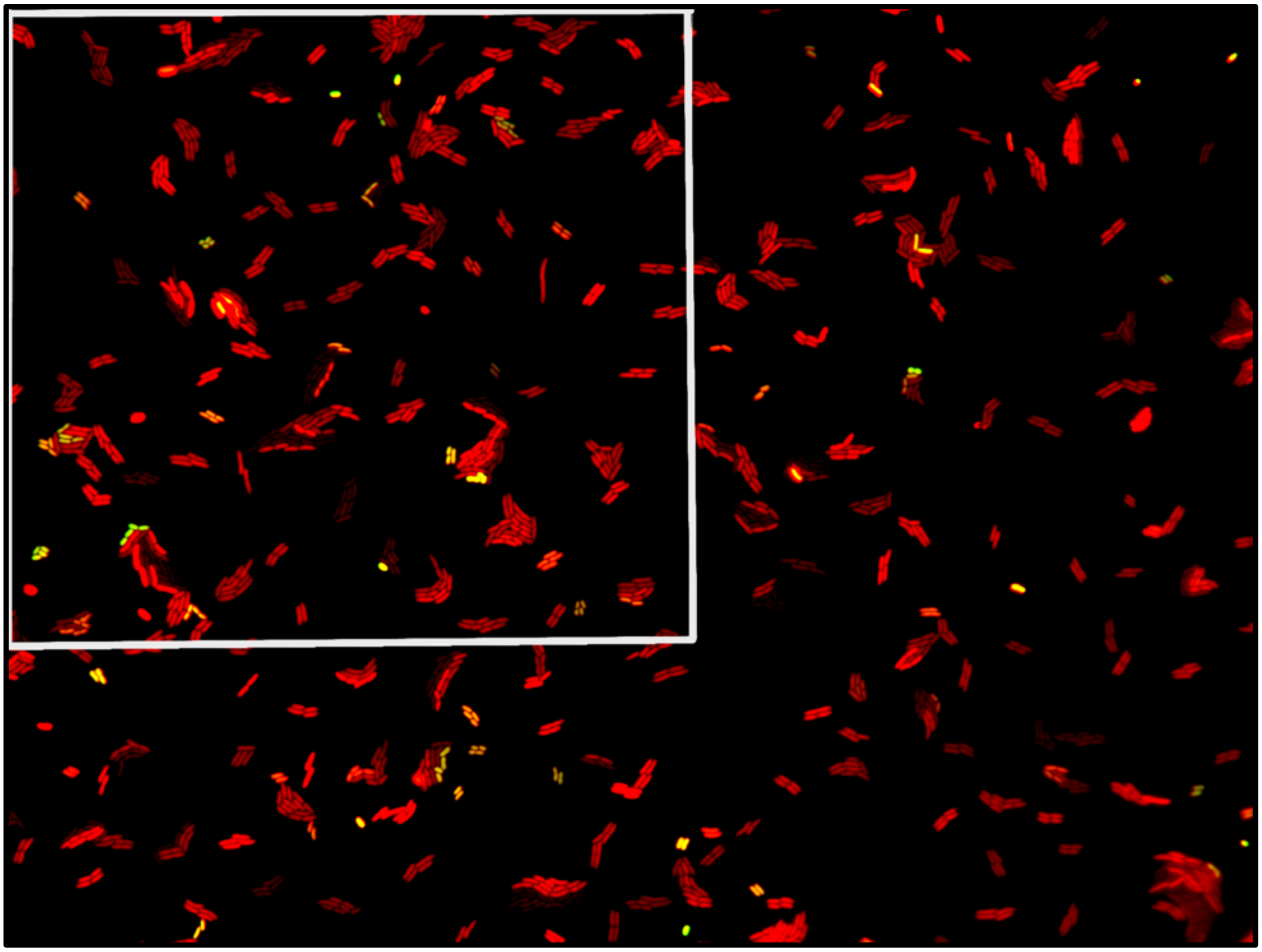

Fig S5. (t) Image1, Fluorescent.

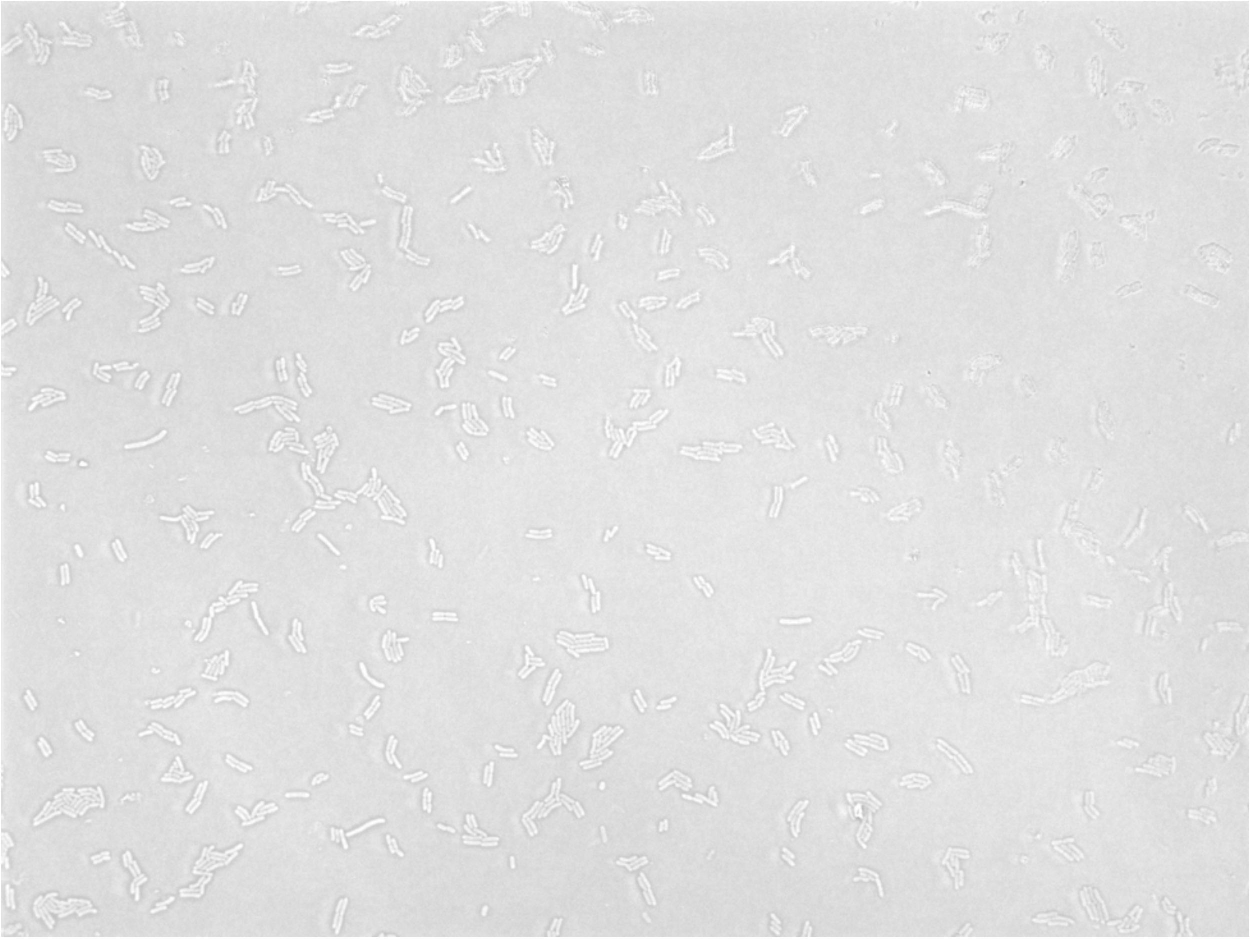

Fig S5. (u) Image2, Brightfield.

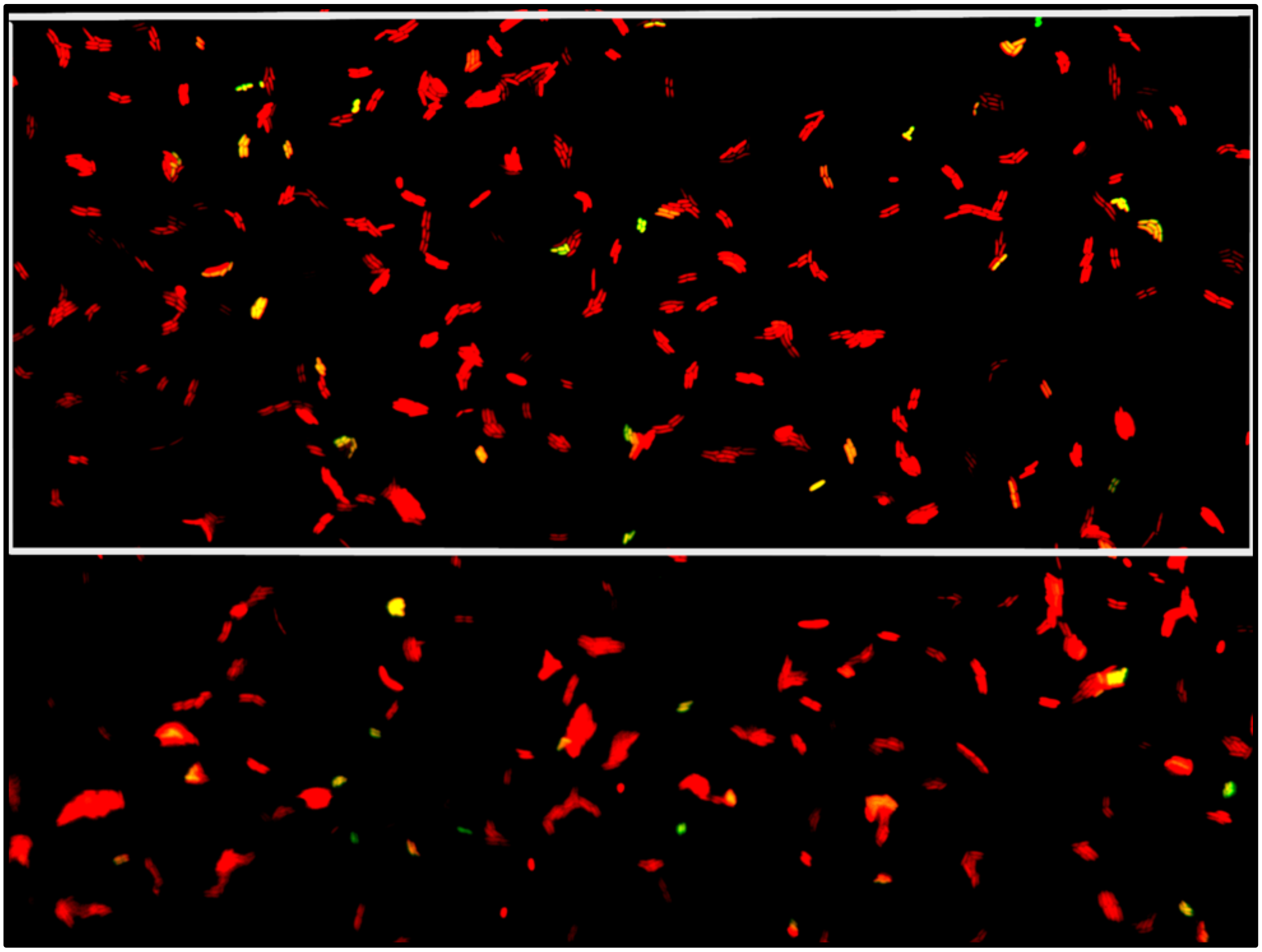

Fig S5. (v) Image2, Fluorescent.

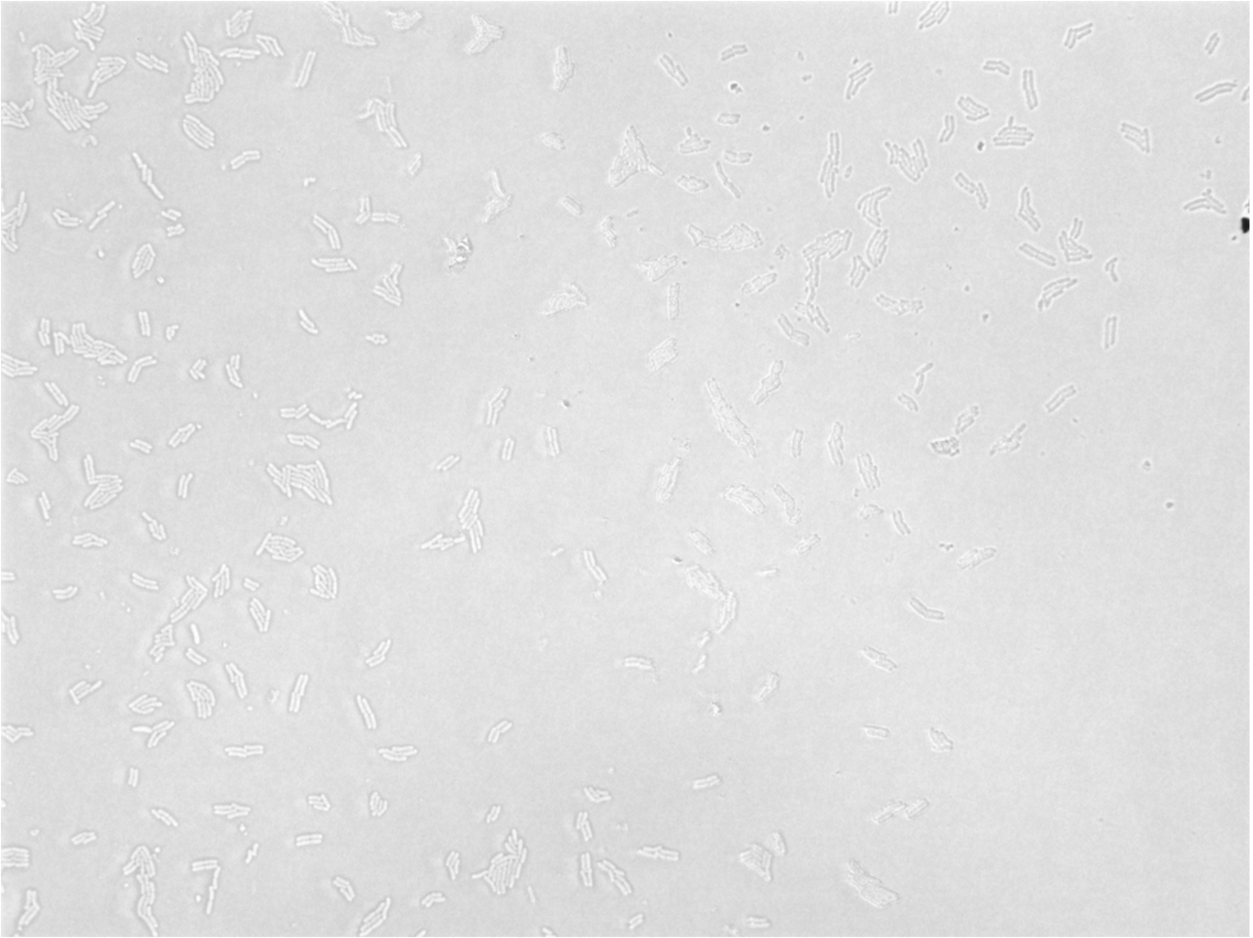

Fig S5. (w) Image3, Brightfield.

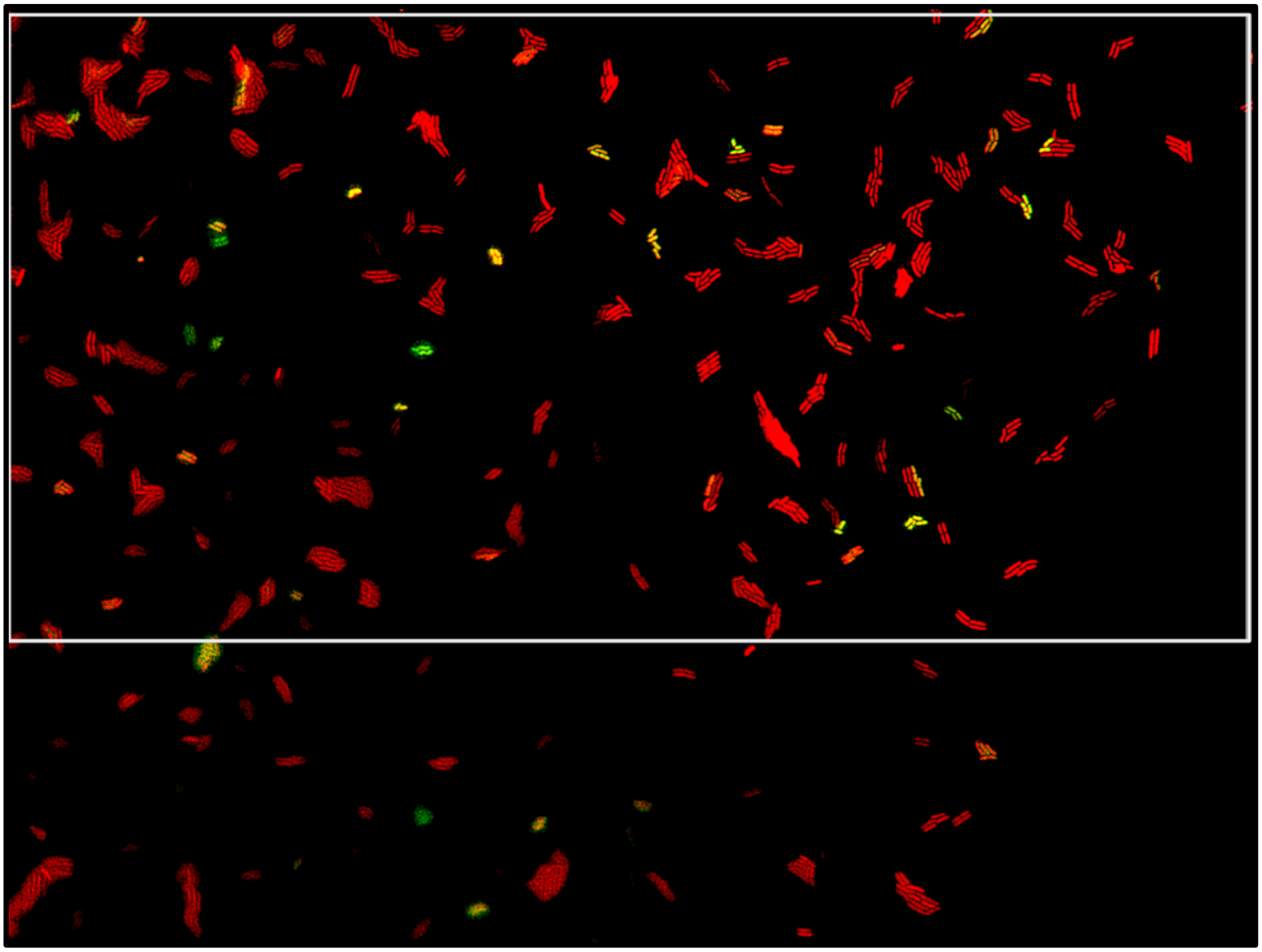

Fig S5. (x) Image3, Fluorescent.

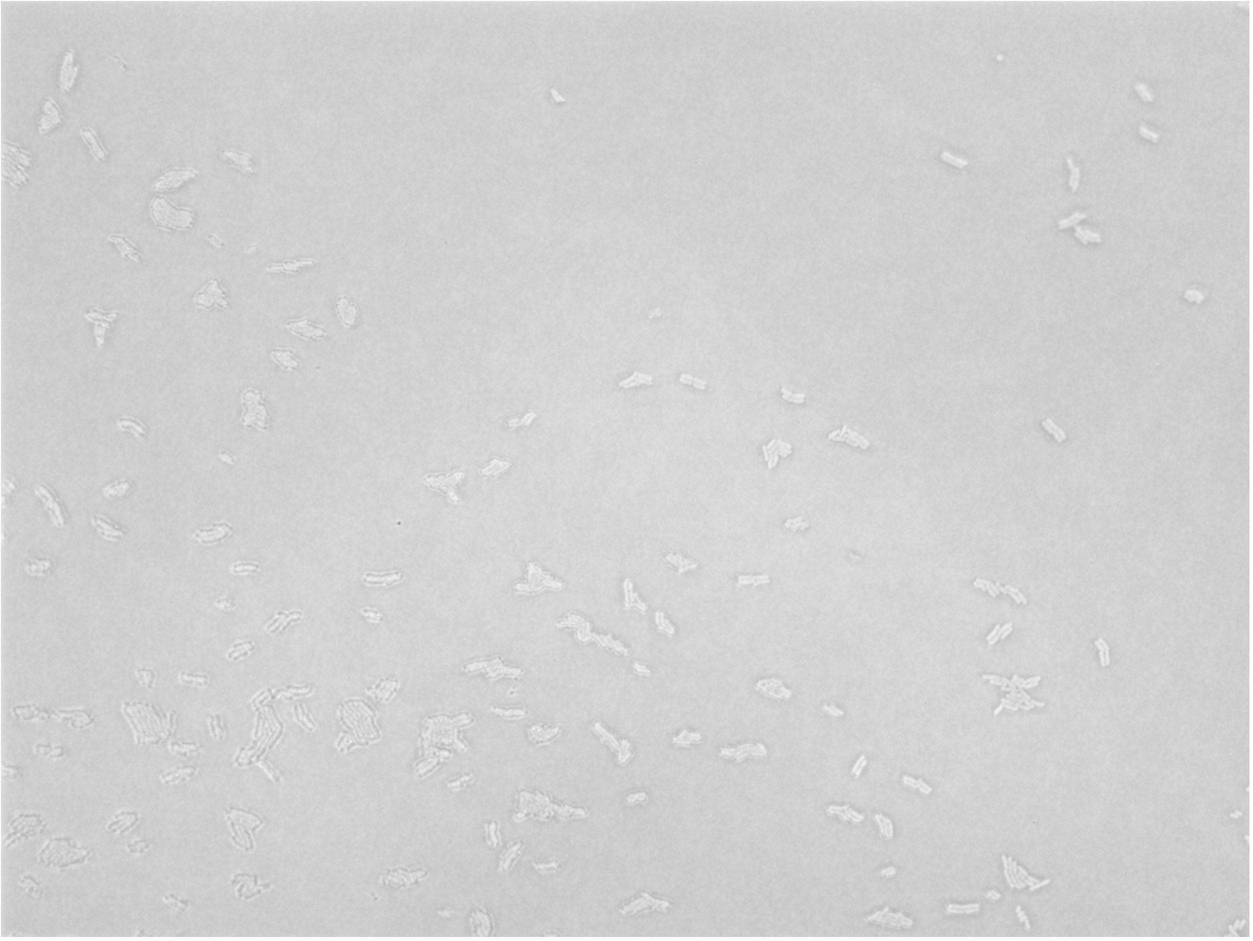

Fig S5. (y) Image4, Brightfield.

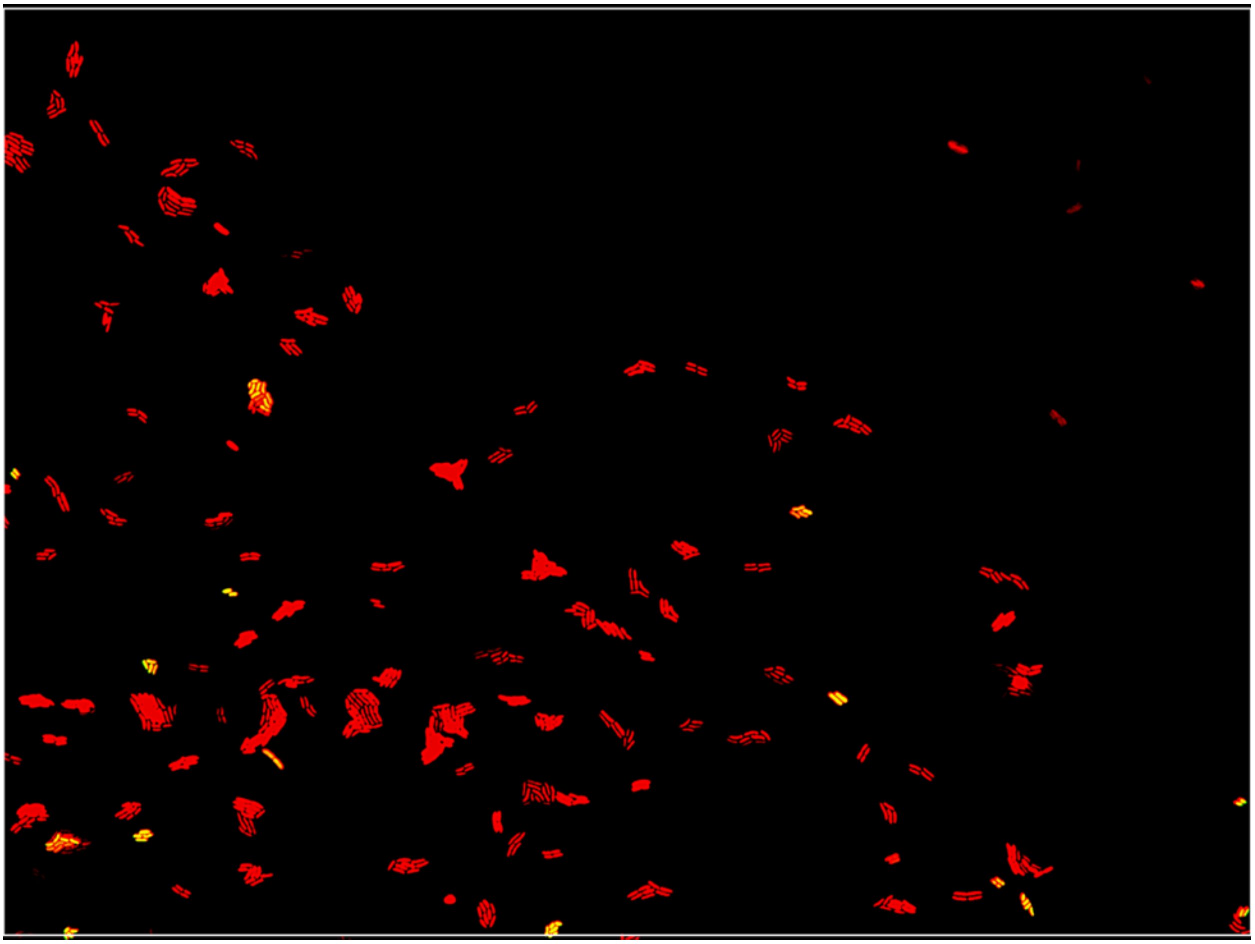

Fig S5. (z) Image4, Fluorescent.

Figure S5. Images used to count T3SS + and T3SS- cells in Extended Fig. 3d. Fig. S5 (a-r) are *Salmonella* and *C. albicans* SC5314 cultures and Fig. S5 (s-z) are *Salmonella* only cultures. *C. albicans* brightfield, STm T3SS- red and STm T3SS^{+} green. Section of image selected for counting (in focus region) is marked.

Supplementary References

1. Miao, J. *et al.* Glycogen Metabolism in Candida albicans Impacts Fitness and Virulence during Vulvovaginal and Invasive Candidiasis. *MBio* **14**, e0004623 (2023).
2. Liu, J. *et al.* Rapid Hypothesis Testing in Candida albicans Clinical Isolates Using a

Cloning-Free, Modular, and Recyclable System for CRISPR-Cas9 Mediated Mutant and Revertant Construction. *Microbiol Spectr* **10**, e0263021 (2022).

3. Nakayama, H. *et al.* Tetracycline-regulatable system to tightly control gene expression in the pathogenic fungus Candida albicans. *Infect. Immun.* **68**, 6712–6719 (2000).
4. Gietz, D., St Jean, A., Woods, R. A. & Schiestl, R. H. Improved method for high efficiency transformation of intact yeast cells. *Nucleic Acids Res.* **20**, 1425 (1992).
5. Znaidi, S. *et al.* The zinc cluster transcription factor Tac1p regulates PDR16 expression in Candida albicans. *Mol. Microbiol.* **66**, 440–452 (2007).
6. Barthel, M. *et al.* Pretreatment of mice with streptomycin provides a Salmonella enterica serovar Typhimurium colitis model that allows analysis of both pathogen and host. *Infect. Immun.* **71**, 2839–2858 (2003).
7. Weening, E. H. *et al.* The Salmonella enterica serotype Typhimurium lpf, bcf, stb, stc, std, and sth fimbrial operons are required for intestinal persistence in mice. *Infect. Immun.* **73**, 3358–3366 (2005).
8. Devlin, J. R. *et al.* Salmonella enterica serovar Typhimurium chitinases modulate the intestinal glycome and promote small intestinal invasion. *PLoS Pathog.* **18**, e1010167 (2022).
9. Raffatellu, M. *et al.* Lipocalin-2 resistance confers an advantage to Salmonella enterica serotype Typhimurium for growth and survival in the inflamed intestine. *Cell Host Microbe* **5**, 476–486 (2009).
10. Lin, D., Rao, C. V. & Slauch, J. M. The Salmonella SPI1 type three secretion system responds to periplasmic disulfide bond status via the flagellar apparatus and the RcsCDB system. *J. Bacteriol.* **190**, 87–97 (2008).
11. Zhang, S. *et al.* The Salmonella enterica serotype typhimurium effector proteins SipA, SopA, SopB, SopD, and SopE2 act in concert to induce diarrhea in calves. *Infect. Immun.* **70**, 3843–3855 (2002).

12. Hoiseth, S. K. & Stocker, B. A. Aromatic-dependent Salmonella typhimurium are non-virulent and effective as live vaccines. *Nature* **291**, 238–239 (1981).

13. Knodler, L. A., Bertero, M., Yip, C., Strynadka, N. C. J. & Steele-Mortimer, O. Structure-based mutagenesis of SigE verifies the importance of hydrophobic and electrostatic residues in type III chaperone function. *Mol. Microbiol.* **62**, 928–940 (2006).

14. Knodler, L. A., Winfree, S., Drecktrah, D., Ireland, R. & Steele-Mortimer, O. Ubiquitination of the bacterial inositol phosphatase, SopB, regulates its biological activity at the plasma membrane. *Cell. Microbiol.* **11**, 1652–1670 (2009).

15. Cooper, K. G. *et al.* Activation of Akt by the bacterial inositol phosphatase, SopB, is wortmannin insensitive. *PLoS One* **6**, e22260 (2011).

16. Hockenberry, A. M. *et al.* Microbiota-derived metabolites inhibit Salmonella virulent subpopulation development by acting on single-cell behaviors. *Proc. Natl. Acad. Sci. U. S. A.* **118**, e2103027118 (2021).

17. Porwollik, S. *et al.* Defined single-gene and multi-gene deletion mutant collections in Salmonella enterica sv Typhimurium. *PLoS One* **9**, e99820 (2014).

18. Rahman, D., Mistry, M., Thavaraj, S., Challacombe, S. J. & Naglik, J. R. Murine model of concurrent oral and vaginal Candida albicans colonization to study epithelial host–pathogen interactions. *Microbes Infect.* **9**, 615–622 (2007).

19. Gillum, A. M., Tsay, E. Y. & Kirsch, D. R. Isolation of the Candida albicans gene for orotidine-5’-phosphate decarboxylase by complementation of S. cerevisiae ura3 and E. coli pyrF mutations. *Mol. Gen. Genet.* **198**, 179–182 (1984).

20. Drecktrah, D. *et al.* Dynamic behavior of Salmonella-induced membrane tubules in epithelial cells. *Traffic* **9**, 2117–2129 (2008).

21. Reuss, O., Vik, A., Kolter, R. & Morschhäuser, J. The SAT1 flipper, an optimized tool for gene disruption in Candida albicans. *Gene* **341**, 119–127 (2004).
