# Supplementary material for: Commensal yeast promotes Salmonella Typhimurium virulence
Source: Nature. 2025 Sep 3;645(8082):1002–10. doi: 10.1038/s41586-025-09415-y (PMC12460169; doi:10.1038/s41586-025-09415-y)
Supplement: Supplementary file 2 — Reporting Summary [file 41586_2025_9415_MOESM2_ESM.pdf]

nature portfolio

Corresponding author(s): Judith Behnsen

Last updated by author(s): 6/29/2025

Reporting Summary

Nature Portfolio wishes to improve the reproducibility of the work that we publish. This form provides structure for consistency and transparency in reporting. For further information on Nature Portfolio policies, see our Editorial Policies and the Editorial Policy Checklist.

Statistics

For all statistical analyses, confirm that the following items are present in the figure legend, table legend, main text, or Methods section.

n/a	Confirmed	
☐	☒	The exact sample size (*n*) for each experimental group/condition, given as a discrete number and unit of measurement
☐	☒	A statement on whether measurements were taken from distinct samples or whether the same sample was measured repeatedly
☐	☒	The statistical test(s) used AND whether they are one- or two-sided<br>*Only common tests should be described solely by name; describe more complex techniques in the Methods section.*
☐	☒	A description of all covariates tested
☐	☒	A description of any assumptions or corrections, such as tests of normality and adjustment for multiple comparisons
☐	☒	A full description of the statistical parameters including central tendency (e.g. means) or other basic estimates (e.g. regression coefficient) AND variation (e.g. standard deviation) or associated estimates of uncertainty (e.g. confidence intervals)
☐	☒	For null hypothesis testing, the test statistic (e.g. *F*, *t*, *r*) with confidence intervals, effect sizes, degrees of freedom and *P* value noted<br>*Give P values as exact values whenever suitable.*
☒	☐	For Bayesian analysis, information on the choice of priors and Markov chain Monte Carlo settings
☒	☐	For hierarchical and complex designs, identification of the appropriate level for tests and full reporting of outcomes
☒	☐	Estimates of effect sizes (e.g. Cohen's *d*, Pearson's *r*), indicating how they were calculated

Our web collection on statistics for biologists contains articles on many of the points above.

Software and code

Policy information about availability of computer code

Data collection	No custom code was used in this study.
Data analysis	No custom code was used in this study.

For manuscripts utilizing custom algorithms or software that are central to the research but not yet described in published literature, software must be made available to editors and reviewers. We strongly encourage code deposition in a community repository (e.g. GitHub). See the Nature Portfolio guidelines for submitting code & software for further information.

Data

Policy information about availability of data

All manuscripts must include a data availability statement. This statement should provide the following information, where applicable:

- Accession codes, unique identifiers, or web links for publicly available datasets
- A description of any restrictions on data availability
- For clinical datasets or third party data, please ensure that the statement adheres to our policy

Raw sequence reads have been deposited at NCBI Sequence Read Archive under project PRJNA1143068, PRJNA1255633, and PRJNA1285498 and the UNITE (v.8.2) database64 was used for all microbiome analysis.

Research involving human participants, their data, or biological material

Policy information about studies with human participants or human data. See also policy information about sex, gender (identity/presentation), and sexual orientation and race, ethnicity and racism.

Reporting on sex and gender	Human research participants is not applicable to this study
Reporting on race, ethnicity, or other socially relevant groupings	Human research participants is not applicable to this study
Population characteristics	Human research participants is not applicable to this study
Recruitment	Human research participants is not applicable to this study
Ethics oversight	Human research participants is not applicable to this study

Note that full information on the approval of the study protocol must also be provided in the manuscript.

Field-specific reporting

Please select the one below that is the best fit for your research. If you are not sure, read the appropriate sections before making your selection.

☒ Life sciences ☐ Behavioural & social sciences ☐ Ecological, evolutionary & environmental sciences

For a reference copy of the document with all sections, see nature.com/documents/nr-reporting-summary-flat.pdf

Life sciences study design

All studies must disclose on these points even when the disclosure is negative.

Sample size	No sample-size calculation was performed. Sample sizes were chosen based on past experience using the methods described (see Santus et al, Nature Microbiology 2022; Behnsen et al, Immunity 2014; Raffatellu et al, Cell Host Microbe 2009; Deriu et al 2013, Cell Host Microbe). Each in vitro experiment was repeated with at least 3 individual biological replicates. Mouse experiments were performed with 2-6 mice per group and repeated for a minimal number of 5 mice per experimental condition.
Data exclusions	As mice are not a natural host for C. albicans, some mice did not maintain C. albicans cecum colonization during the experiment. These mice were excluded from further analysis.
Replication	Each in vitro experiment was performed at least in triplicate. Mouse experiments were performed 2-4 times. All replication attempts performed under the same conditions were successful.
Randomization	Mice were received from vendors and randomly assigned to experimental groups. For in vitro experiments, samples could not be randomized, as each was a specific experimental condition that needed to be defined.
Blinding	Blinding was not performed. No measurements sensitive to subjective interpretation were performed.

Reporting for specific materials, systems and methods

We require information from authors about some types of materials, experimental systems and methods used in many studies. Here, indicate whether each material, system or method listed is relevant to your study. If you are not sure if a list item applies to your research, read the appropriate section before selecting a response.

Materials & experimental systems

n/a	Involved in the study	
☐	☒	Antibodies
☐	☒	Eukaryotic cell lines
☒	☐	Palaeontology and archaeology
☐	☒	Animals and other organisms
☒	☐	Clinical data
☒	☐	Dual use research of concern
☒	☐	Plants

Methods

n/a	Involved in the study	
☒	☐	ChIP-seq
☒	☐	Flow cytometry
☒	☐	MRI-based neuroimaging

Antibodies

Antibodies used	Goat anti-Rabbit IgG (H+L) Cross-Adsorbed ReadyProbes™ Secondary Antibody, Alexa Fluor™ 594; Invitrogen, Cat# R37117, Lot# 1938330<br>FITC-labeled rabbit anti-fungal antibody; Meridian Life Science, Cat# B65411F, Lot# 2D11517
Validation	Both antibodies used in this study were validated by the manufacturers. Comprehensive validation statements, alongside relative details such as species reactivity, applications, and supporting citations, are accessible through the provided website links. Essential information pertaining to the antibodies employed in this study are outlined below:<br><br>Goat anti-Rabbit IgG (H+L) Cross-Adsorbed ReadyProbes™ Secondary Antibody, Alexa Fluor™ 594; Invitrogen<br>Species reactivity: Rabbit<br>Application (in this study): Immunocytochemistry (ICC/IF)<br>Validation statements and other details (including citations): https://www.thermofisher.com/antibody/product/Goat-anti-Rabbit-IgG-H-L-Cross-Adsorbed-Secondary-Antibody-Polyclonal/R37117<br><br>FITC-labeled rabbit anti-fungal antibody; Meridian Life Science<br>Application (in this study): Immunocytochemistry (ICC/IF)<br>Validation statements and other details (including citations): https://www.meridianbioscience.com/uploads/ls-uploads/coa/B65411F.pdf

Eukaryotic cell lines

Policy information about cell lines and Sex and Gender in Research

Cell line source(s)	T84 colonic epithelial cell line; ATCC Cat# CCL-248; human carcinoma cell line derived from a lung metastasis of a colon carcinoma in a 72-year-old male; RRID:CVCL_0555<br><br>C2BBe1 [clone of Caco-2]; ATCC Cat# CCL-2102; enterocytes isolated from the large intestine (colon) of a patient with colorectal adenocarcinoma; RRID:CVCL_1096
Authentication	Cell lines used were not authenticated
Mycoplasma contamination	Both T84 and C2BBe1 cell lines tested negative for mycoplasma contamination
Commonly misidentified lines<br>(See ICLAC register)	Commonly misidentified cell lines were not used

Animals and other research organisms

Policy information about studies involving animals; ARRIVE guidelines recommended for reporting animal research, and Sex and Gender in Research

Laboratory animals	conventional C57BL/6 mice 8-9 weeks old ; gnotobiotic Swiss Webster mice 12-15 weeks old ; CBA/J mice 8-9 weeks old, gnotobiotic C57BL/6 mice 8-10 week old
Wild animals	The study did not involve wild animals.
Reporting on sex	Both male and female mice were used in the study.
Field-collected samples	The study did not involve samples collected from the field.
Ethics oversight	All animal experiments were reviewed and approved by the Institutional Animal Care and Use Committee at the University of Illinois Chicago (protocols 17-045, 20-016 and 22-192).

Note that full information on the approval of the study protocol must also be provided in the manuscript.

Plants

Seed stocks	N/A- This study do not involve plants
Novel plant genotypes	N/A- This study do not involve plants
Authentication	N/A- This study do not involve plants
