# Supplementary material for: Commensal yeast promotes Salmonella Typhimurium virulence
Source: Nature. 2025 Sep 3;645(8082):1002–10. doi: 10.1038/s41586-025-09415-y (PMC12460169; doi:10.1038/s41586-025-09415-y)
Supplement: Supplementary file 4 — Supplementary Tables 1–13 [file 41586_2025_9415_MOESM4_ESM.zip › 2024-07-15633B-s4/Supplementary Table legends.docx]

Supplementary Tables 1-13

Table S1.

ITS sequencing results of fecal samples before and after *Salmonella* infection.

Table S2.

RNAseq data showing differentially regulated *Salmonella* genes in co-culture with *C. albicans* ATCC compared to *Salmonella* alone.

Table S3.

KEGG analysis of pathways differentially regulated in *Salmonella* co-culture with *C. albicans* ATCC compared to *Salmonella* alone.

Table S4.

Amino acid levels in the supernatant of *Salmonella* and *C. albicans* cultures.

Table S5.

Amino acid levels in the cecum content of mice infected with *Salmonella*, *C. albicans*, or co-infected mice 48h p.i.

Table S6.

Amino acid levels in the cecum content of mice infected with *Salmonella*, *C. albicans*, or co-infected mice 24h p.i.

Table S7.

Amino acid levels in the cecum content of Germ-free mice uninfected or infected with *Salmonella* and *C. albicans*.

Table S8.

Amino acid levels in the cecum content of ASF mice uninfected or infected with *Salmonella* and *C. albicans*.

Table S9.

ITS sequencing results of fecal samples after *Salmonella* or *Salmonella* and *C. albicans* infection.

Table S10.

16S sequencing results of fecal samples after *Salmonella* or *Salmonella* and *C. albicans* infection.

Table S11.

Real-time PCR analysis of genes encoding for host inflammatory response from the cecum tissue of Uninfected or Uninfected with L-arginine treated mice 24h and 48h p.i. Data is from 2 independent experiments.

Table S12.

Real-time PCR analysis of genes encoding for host inflammatory response from the cecum tissue of *Salmonella* infected or *Salmonella* infected with L-arginine treated mice 48h p.i. Data is from 2 independent experiments.

Table S13.

Amino acid levels in the cecum content of uninfected or *Salmonella* infected mice in the presence or absence of L-arginine.
